# Microbial differences between dental plaque and historic dental calculus are related to oral biofilm maturation stage

**DOI:** 10.1186/s40168-019-0717-3

**Published:** 2019-07-06

**Authors:** Irina M. Velsko, James A. Fellows Yates, Franziska Aron, Richard W. Hagan, Laurent A. F. Frantz, Louise Loe, Juan Bautista Rodriguez Martinez, Eros Chaves, Chris Gosden, Greger Larson, Christina Warinner

**Affiliations:** 1 The Palaeogenomics and Bio-Archaeology Research Network, Research Laboratory for Archaeology and the History of Art, University of Oxford, Oxford, OX1 3QY UK; 2 Department of Archaeogenetics, Max Planck Institute for the Science of Human History, 07745 Jena, Germany; 3 School of Biological and Chemical Sciences, Queen Mary University of London, London, E1 4NS UK; 4 Heritage Burial Services, Oxford Archaeology, Oxford, OX2 0ES UK; 5 Dental Office Dr. Juan Bautista Rodriguez, Pozo Alcon, Jaén Spain; 6 Department of Periodontics, University of Oklahoma Health Sciences Center, Oklahoma City, 73117 OK USA; 7 Department of Anthropology, University of Oklahoma, Norman, OK 73019 USA; 8 Current address: Pinellas Dental Specialties, Largo, FL 33776 USA

**Keywords:** Ancient dental calculus, Oral microbiome, Metagenomics, Metaproteomics, Periodontal disease

## Abstract

**Background:**

Dental calculus, calcified oral plaque biofilm, contains microbial and host biomolecules that can be used to study historic microbiome communities and host responses. Dental calculus does not typically accumulate as much today as historically, and clinical oral microbiome research studies focus primarily on living dental plaque biofilm. However, plaque and calculus reflect different conditions of the oral biofilm, and the differences in microbial characteristics between the sample types have not yet been systematically explored. Here, we compare the microbial profiles of modern dental plaque, modern dental calculus, and historic dental calculus to establish expected differences between these substrates.

**Results:**

Metagenomic data was generated from modern and historic calculus samples, and dental plaque metagenomic data was downloaded from the Human Microbiome Project. Microbial composition and functional profile were assessed. Metaproteomic data was obtained from a subset of historic calculus samples. Comparisons between microbial, protein, and metabolomic profiles revealed distinct taxonomic and metabolic functional profiles between plaque, modern calculus, and historic calculus, but not between calculus collected from healthy teeth and periodontal disease-affected teeth. Species co-exclusion was related to biofilm environment. Proteomic profiling revealed that healthy tooth samples contain low levels of bacterial virulence proteins and a robust innate immune response. Correlations between proteomic and metabolomic profiles suggest co-preservation of bacterial lipid membranes and membrane-associated proteins.

**Conclusions:**

Overall, we find that there are systematic microbial differences between plaque and calculus related to biofilm physiology, and recognizing these differences is important for accurate data interpretation in studies comparing dental plaque and calculus.

**Electronic supplementary material:**

The online version of this article (10.1186/s40168-019-0717-3) contains supplementary material, which is available to authorized users.

## Background

Dental calculus is a mineralized oral plaque biofilm that preserves biomolecules such as DNA and protein over long periods of time in the archeological record [1–7], and as such, it has the potential to offer insight into human microbiome evolution. Most clinical oral microbiome studies focus on dental plaque rather than calculus, in part because it is easier to study, it represents a living (and thus active) biofilm, and because dental plaque is directly responsible for oral pathology [8]. Comparatively less is known about the structure and formation of dental calculus, and studies of modern calculus are additionally hampered by the fact that deposits are smaller and less prevalent in living populations practicing tooth brushing and other forms of active oral hygiene [9, 10]. Although calculus forms from dental plaque, microbial profile differences have been noted between historic calculus and modern dental plaque [1, 2], but reasons for these differences, such as degree of biofilm maturation, have not yet been sufficiently investigated. In order to advance the studies of oral microbiome evolution, it is necessary to understand the basis of observed differences between the microbial profiles of ancient dental calculus and modern dental plaque.

Oral biofilm development and maturation have been characterized both in vitro and in vivo, and the microbial succession of dominant species over hours and days is well-described [11–14]. This has led to the classification of some oral taxa as “early colonizers” and others as “late colonizers” [15], and progressive taxonomic shifts are correlated with structural and resource changes in the biofilm through time. Early colonizers, for example, are typically facultative anaerobes and often saccharolytic, feeding primarily on salivary mucins and other glycoproteins [16]. During the course of biofilm growth and maturation, oxygen is progressively depleted and proteolytic obligate anaerobes, including methanogens and sulfate-reducers, rise in abundance [16], thus resulting in the formation of a fully mature oral biofilm profile. This mature community is likely the biofilm stage historically preserved in dental calculus. The microbial profiles of ancient dental calculus often contain high proportions of proteolytic obligate anaerobes, including *Tannerella*, *Porphyromonas*, *Methanobrevibacter*, and *Desulfobulbus* [1, 2], and therefore resemble a fully mature oral biofilm. However, today, frequent removal of supragingival plaque by tooth brushing and professional dental cleaning prevents the biofilm from fully maturing. This potentially makes a direct comparison with contemporary dental plaque, especially plaque regularly disrupted by oral hygiene regimens, more complicated.

Several species that characterize mature oral biofilms are strongly associated with oral disease in dental plaque. Socransky et al. [17] assessed the subgingival plaque bacterial profiles by cluster analysis and described 5 now-classic microbial complexes, named by color: yellow, purple, green, orange, and red. They further determined that the orange, and especially red, complexes were associated with clinical parameters of periodontal disease. Consequently, the trio of anaerobic, proteolytic, and asaccharolytic species known as the “red complex”—*Porphyromonas gingivalis*, *Tannerella forsythia*, and *Treponema denticola*—have come to be widely regarded as specific indicators of oral disease [18–22], despite being more abundant in mature plaque generally. Whether these species are specifically representative of disease-associated biofilms in ancient dental calculus is not yet clear.

Here, we compare microbial community profiles among modern dental plaque, modern dental calculus, and historic dental calculus in order to establish characteristic microbial profile differences between plaque and calculus, as well as between calculus samples before and after the twentieth century modernization efforts in oral hygiene, sanitation, and medicine. In addition, we investigated the differences in microbial profiles between calculus from healthy tooth sites and diseased tooth sites, to understand whether the microbial species distinctions between healthy tooth biofilm profiles and disease tooth biofilm profiles reported in dental plaque are also present in calculus. We found that species profiles of plaque and calculus are similar, but with notable exceptions related to biofilm maturity, while disease-associated species are generally more abundant in calculus despite tooth health status. Finally, we demonstrate that integration of taxonomic, proteomic, and metabolomic profiles of historic calculus can reveal preservation patterns that would not be clear from single-omics profiling.

## Results

### Authentication of a preserved oral biofilm in calculus samples

Overall, source estimation analysis indicates good oral microbiome preservation across the datasets. SourceTracker analysis of historic and modern calculus samples (Additional file 1: Figure S1) demonstrated that both sample groups have a predominantly oral microbial signature. The strong gut signature in several samples is characteristic of calculus and a known artifact of using QIIME to classify filtered 16S rRNA metagenomic reads, whereby several characteristically oral taxa (e.g., *Lactobacillales* spp.) are systematically misclassified as close relatives in the gut [23]. Additionally, the strong “unknown” signature in several samples likely stems from the presence of similar taxa found in both oral and gut source samples, such as *Methanobrevibacter* spp. and *Tisseriellaceae* [5]. Prior to analysis, historic calculus sample sequence reads were assessed for the presence of damage patterns that characterize ancient DNA. MapDamage plots of reads that mapped to the genome of the oral bacterium *Tannerella forsythia*, a species both prevalent and abundant in historic dental calculus, and to the human genome display elevated C to T transitions at molecule ends (Additional file 1: Figure S2), indicative of deamination, a pattern typical of authentic ancient DNA.

### Dental calculus and plaque biofilm communities are distinct

**Fig. 1:**
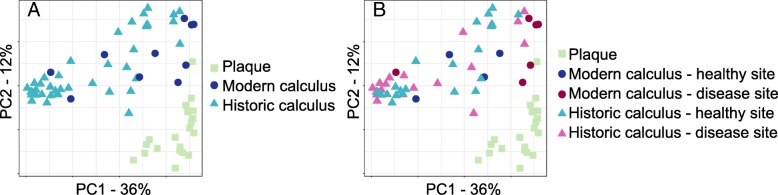
Dental plaque and dental calculus contain distinct microbial communities. **a** Principal component analysis (PCA) clusters modern dental plaque distinctly from modern and historic dental calculus, while calculus samples do not separate by time period. **b** PCA clustering of calculus samples is not related to health status

To investigate whether there are systematic differences in microbial communities between modern dental plaque and ancient and modern dental calculus, we compared the communities found in modern supra- and subgingival plaque from the Human Microbiome Project (HMP), modern calculus, and 200-year-old historic calculus (Table 1). A principal component analysis (PCA) of the species profiles of each group clustered the modern plaque samples distinctly from the calculus samples, while the modern and historic calculus samples were intermixed (Fig. 1a), suggesting that the differences between microbial profiles of plaque and calculus are more pronounced than between historic and modern calculus. We investigated whether periodontal disease or caries on the sampled tooth explained the clustering of the calculus samples, but no clustering was observed based on the presence or absence of disease in historic samples (Fig. 1b). The modern samples appear to cluster by health status with the exception of a single disease site sample, although additional samples are needed to confirm this trend. Further, the distribution of calculus sample points is not related to the sequencing depth or sequencing run. Among HMP samples, separation was observed for supra- or subgingival plaque (Additional file 1: Figure S3).

**Table 1:** Sample demographics

	Plaque	Modern calculus	Historic calculus
Source	HMP	This study	This study
N	20	10	43
Periodontal disease^*^			
Yes	0	6	18
No	20	4	25
Caries^*^			
Yes	0	10	8
No	20	0	35

^*^On the tooth/teeth sampled. Detailed sample metadata is presented in Additional file 2: Table S1

**Fig. 2:**
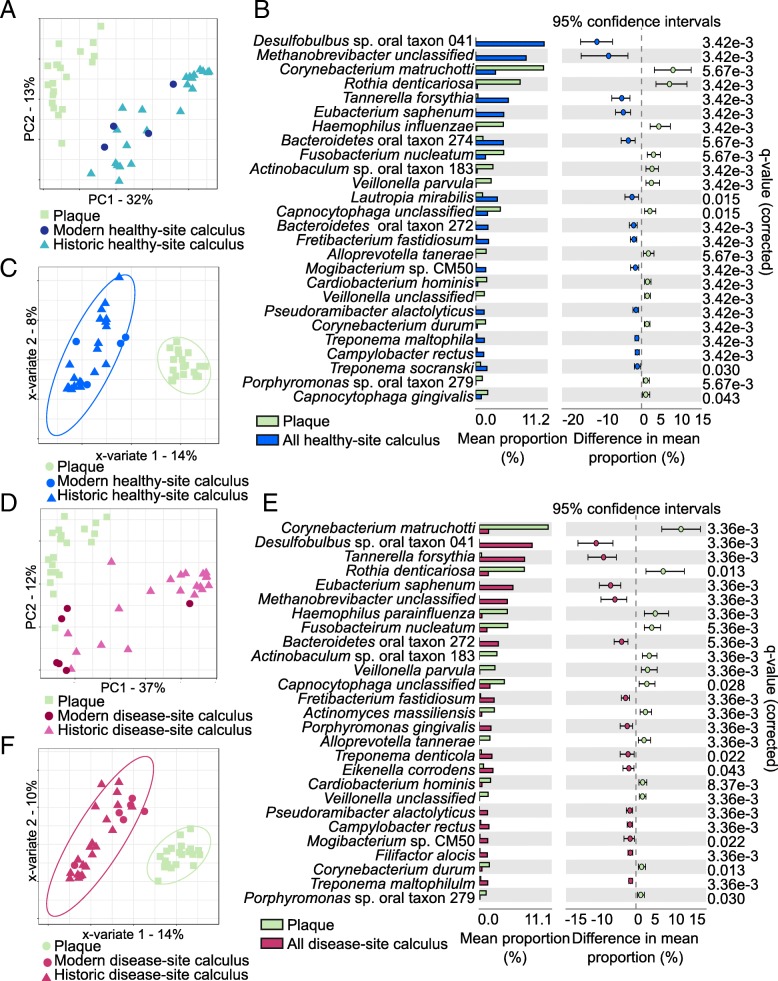
Microbial profile differences between plaque and calculus of differing health status. **a** Principal component analysis (PCA) clusters plaque and healthy site calculus distinctly. **b** Distinct species are significantly more abundant in plaque and healthy site calculus. **c** Microbial profile differences between plaque and all healthy site calculus are sufficient for discrimination of sample types by sparse partial least squares-discriminant analysis (sPLS-DA). Ellipses indicate 95% confidence intervals. **d** PCA clusters plaque and disease site calculus distinctly. **e** Distinct species are significantly more abundant in plaque and disease site calculus. **f** Microbial profile differences between plaque and all disease site calculus are sufficient for discrimination of sample types by sPLS-DA. Ellipses indicate 95% confidence intervals

Because the HMP samples were collected from patients with no overt evidence of periodontal disease or caries, we investigated the differences between healthy tooth site plaque and healthy tooth site calculus by performing a PCA using all HMP samples and only healthy site calculus from both modern and historic sources. Again, the HMP samples clustered distinctly from the calculus samples, with no separation of modern and historic calculus (Fig. 2a), but were not significantly different by adonis test. Twenty-six species were significantly (*q* ≤ 0.05, effect size ≥ 1) differentially abundant between plaque and calculus samples (Fig. 2b), with 13 more abundant in plaque and 13 more abundant in calculus. Many of the taxa with higher abundance in calculus are “late colonizers” (i.e., *Desulfobulbus*, *Methanobrevibacter*, *Tannerella*) associated in modern patients with mature biofilms and periodontal disease. Sparse partial squares-discriminant analysis (sPLS-DA) of the plaque and healthy site calculus samples allowed us to visualize how informative our sample sets are for classifying plaque and calculus based on microbial profile and to select the species that contribute most to classifying each sample. The two groups clustered tightly in the PLS-DA (Fig. 2c) indicating that they have distinct profiles, which was confirmed by the low classification balanced error rate (BER) (< 0.0001, Additional file 2: Table S3), and many of the same species that contribute to classification are differentially abundant (Additional file 1: Figure S4A).

We then tested whether plaque and calculus from a tooth affected by either caries or periodontal disease were equally distinct. A PCA plot indicated that the plaque and calculus samples were clustered mostly distinctly but with a slight overlap between plaque and modern calculus (Fig. 2d), but group separation was not significant by adonis test. Fourteen species were more abundant in diseased tooth site calculus than healthy site plaque, and 13 were more abundant in healthy site plaque (*q* ≤ 0.05, effect size ≥ 1) (Fig. 2e). Of the 14 species with greater abundance in disease site calculus, 11 are also more abundant in healthy site calculus, but other species including *Porphyromonas gingivalis*, *Treponema denticola*, and *Filifactor alocis*, all of which are strongly associated with periodontal disease site plaque [17, 24], are significantly more abundant only when comparing disease site calculus to healthy site plaque. Sparse PLS-DA again demonstrated that our samples are sufficiently informative to classify healthy site plaque and disease site calculus based on microbial profile (Fig. 2f), which was confirmed by the low classification balanced error rate (BER) (< 0.0008, Additional file 2: Table S3), and the species that contribute most to the classification are differentially abundant between the two groups (Additional file 1: Figure S4B). We repeated these analyses comparing healthy site plaque profiles to calculus only affected by periodontal disease (i.e., excluding caries) and found nearly identical trends (Additional file 1: Figure S5A-D).

### Health-associated communities of dental plaque and calculus are distinct

**Fig. 3:**
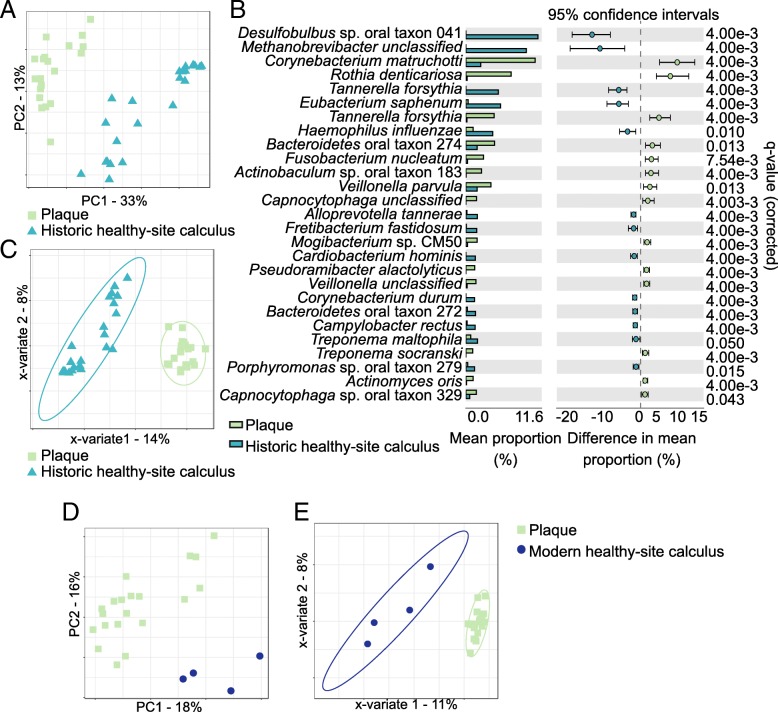
Healthy site calculus microbial profile differs from plaque independent of sample age. **a** Principal component analysis (PCA) of plaque and historic healthy site calculus cluster samples by type. **b** Distinct species are significantly more abundant in plaque and historic healthy site calculus. **c** Microbial profile differences between plaque and historic healthy site calculus are sufficient for discrimination of sample types by sparse partial least squares-discriminant analysis (sPLS-DA). Ellipses indicate 95% confidence intervals. **d** PCA clusters plaque and modern healthy site calculus distinctly. **e** Microbial profile differences between plaque and modern healthy site calculus are sufficient for discrimination of sample types by sPLS-DA. Ellipses indicate 95% confidence intervals

We next explored the microbial profile differences between healthy site plaque and healthy site modern calculus or healthy site historic calculus samples independently to look for time-related differences in microbial communities of the two substrates. Differences would suggest that there are shared properties of health-associated communities that are important for maintaining health, which could be further investigated for understanding host-microbiome interactions that promote health and prevent disease. Both modern and historic calculus samples independently cluster distinctly from modern plaque in PCA plots, even with few modern calculus samples (Fig. 3a, d), supporting that microbial profiles of healthy calculus, both historic and modern, are distinct from plaque, but group separation was not significant by adonis test. Twenty-seven species were significantly differentially abundant between plaque and historic healthy site calculus (*q* ≤ 0.05, effect size ≥ 1) (Fig. 3b), with 13 more abundant in historic healthy site calculus and 14 more abundant in plaque. Most of the species differentially abundant between plaque and healthy site historic calculus are the same as those that are differentially abundant between plaque and all healthy site calculus samples, so the modern calculus profiles fall within the variation of the historic samples. In contrast, no species were significantly differentially abundant between the plaque and modern healthy site calculus; however, this may be because we have only four samples.

Sparse PLS-DA grouped the plaque into a tight cluster compared to the calculus samples (Fig. 3c, e), while the calculus samples, both modern and historic, were more dispersed. In both sPLS-DAs, the BER for plaque was < 0.0001, demonstrating highly accurate sample classification, and the historic healthy site samples had a similarly low BER while the modern healthy site samples were classified less reliably (Additional file 2: Table S3). Many of the species contributing most strongly to the grouping are the same as those that contribute to grouping the calculus samples in our initial plaque vs. all calculus microbial profile comparisons (Additional file 1: Figure S6A,B). When comparing modern plaque profiles to historic healthy site calculus profiles, many of the species contributing most to the classification of the groups are the same as those that are differentially abundant between the two, yet the species with the strongest contributions to the classification are not those with the greatest differential abundance. Several of the species contributing to the classification of both modern and historic healthy site calculus are strongly associated with periodontal disease in modern populations, including the “red complex” members *Porphyromonas gingivalis* and *Tannerella forsythia*, as well as *Filifactor alocis*, an emerging periodontal pathogen [25], again indicating that the presence and abundance of these species in calculus cannot be used as indicators of biofilm pathogenicity.

### Signatures of health and of disease are shared in modern and historic calculus samples

**Fig. 4:**
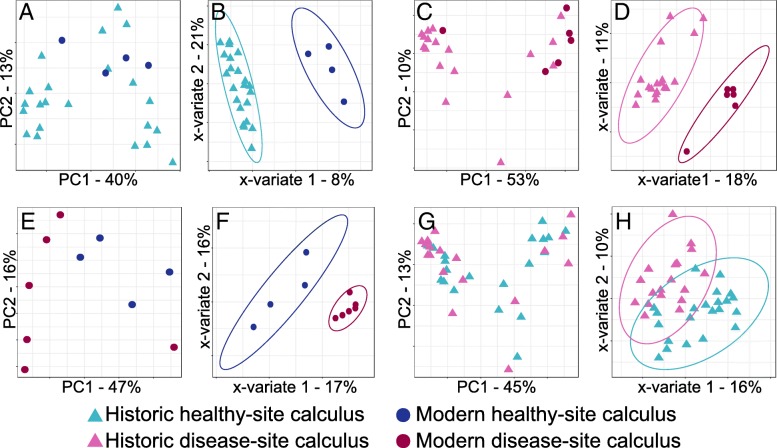
Modern and historic calculus microbial community profiles overlap. **a** Principal component analysis (PCA) does not cluster modern and historic healthy site calculus distinctly. **b** Historic and modern healthy site calculus contain sufficient discriminatory taxa for distinct clustering by sparse partial least squares-discriminant analysis (sPLS-DA). **c** PCA does not cluster modern and historic disease site calculus distinctly. **d** Historic and modern disease site calculus contain sufficient discriminatory taxa for distinct clustering by sPLS-DA. **e** Modern healthy and disease site calculus microbial profiles are not distinctly different and are not separated by PCA. **f** Modern healthy and disease site calculus contain sufficient discriminatory taxa for distinct clustering by sPLS-DA. **g** Historic healthy and disease site calculus microbial profiles are not distinctly different and are not separated by PCA. **h** Modern healthy and disease site calculus do not contain sufficient discriminatory taxa for distinct clustering by sPLS-DA. Ellipses indicate 95% confidence intervals

Since we found that plaque and calculus contain distinct microbial profiles, we tested whether modern and historic calculus microbial profiles are distinct from each other. Although they are the same substrate, the effects of modern hygiene practices such as tooth brushing and fluoridation of drinking water on biofilm development and calculus formation are not well understood. Principal component analyses comparing historic and modern healthy site calculus (Fig. 4a) or historic and modern disease site calculus (Fig. 4c) microbial profiles did not cluster the modern and historic samples distinctly. Only *Campylobacter rectus* was significantly differentially abundant between healthy site historic and modern calculus (Additional file 1: Figure S7A), and no species were significantly differentially abundant between disease site historic and modern calculus. Sparse PLS-DAs clustered historic and modern calculus samples separately for both healthy site and disease site samples (Fig. 4b, d), indicating that the microbial profiles can discriminate health status in modern calculus. However, the BER for historic samples was much smaller than for modern samples in both sPLS-DAs (Additional file 2: Table S3), which may be due to the low modern sample numbers. Several species that contribute most to the classification of modern calculus, both healthy site and disease site, are “early colonizer,” health-associated species within the genera *Actinomyces*, *Streptococcus*, and *Veillonella* (Additional file 1: Figure S7B,C), which are not characteristic of historic calculus samples. Classification of historic calculus samples, both healthy site and disease site, is driven by many of the same species that are differentially abundant in historic calculus compared to plaque (Fig. 2b) and that contribute to the classification of historic samples compared to plaque (Additional file 1: Figure S5D), including *C. rectus*, *Desulfobulbus* sp. oral taxon 041 (aka *Desulfobulbus oralis* [26]), and *F. alocis* (Additional file 1: Figure S7B,C).

### Microbial community differences between health and disease in calculus are poorly resolved

Modern healthy site plaque and periodontal disease site plaque or caries site plaque often contain distinct microbial profiles that are considered signatures of health and disease. We examined modern and historic calculus samples to investigate if the microbial communities of these samples, like plaque, are distinct. Healthy site and periodontal disease site calculus samples did not cluster separately in PCA plots for either modern or historic samples (Fig. 4e, g). Neither historic nor modern calculus samples had significantly differentially abundant species between healthy site and periodontal disease site samples. Sparse PLS-DA clustered modern disease site calculus samples tightly, while healthy site samples were more dispersed but still a distinct cluster (Fig. 4f); however, the BER for both sPLS-DAs (Additional file 2: Table S3) was high, which may be due to the low modern sample numbers and/or high variability. In contrast, sPLS-DA clustering of historic healthy site and periodontal disease site calculus did not tightly or distinctly cluster the groups (Fig. 4h), suggesting the microbial communities of healthy and disease site historic samples are more similar to each other than are modern calculus healthy and disease site communities.

The species that contribute most to the classification of healthy site and disease site calculus largely do not overlap with those species that distinguish calculus from plaque, or healthy site and disease site plaque, as reported in the literature (Additional file 1: Figures S7D, S7E). For example, *Bacteriodetes* oral taxon 274, *Campylobacter gracilis*, and *Pseudopropionibacterium propionicum* distinguish modern healthy site calculus, while *Gemella hemolysans* characterized modern periodontal disease site calculus. While *P. gingivalis* and *T. forsythia* characterized historic periodontal disease site from historic healthy site calculus as they do in modern plaque, additional species that did so are not well-characterized in modern healthy and disease site plaque, such as “early colonizer” *Actinomyces cardiffensis* and *A. timonensis*, as well as several unnamed species in the genera *Bacteroidetes*, *Neisseria*, and *Atopobium* (Additional file 1: Figure S7E). The four historic calculus samples collected from the teeth with both caries and periodontal disease did not appear to have microbial profiles distinct from the samples collected from the teeth with only periodontal disease, as they were distributed throughout the other samples in both the PCA and sPLS-DA plots (Additional file 1: Figure S8).

### Absence of caries-specific microbial profiles in dental calculus

We additionally examined the differences in microbial profiles of historic caries and healthy site calculus. The caries and healthy site samples did not cluster distinctly in PCA (Additional file 1: Figure S9A) nor were there any significantly differentially abundant species between the groups. Healthy site and caries site calculus sample clusters overlapped in sPLS-DA plots (Additional file 1: Figure S9B), and the classification BERs were > 0.1 (Additional file 2: Table S3), indicating that classifying the groups with our data is not reliable. Many of the species that contribute most to the classification of the caries site samples are species that classify historic periodontal disease site from healthy site calculus (Additional file 1: Figure S9C). Three of the five samples with both caries and periodontal disease are the furthest points from the healthy site samples in the sPLS-DA plot (Additional file 1: Figure S9B), so it is likely that the signature of periodontal disease and not of caries is responsible for the clustering and classification.

### Microbial co-exclusion patterns in plaque and calculus reflect biofilm maturity

**Fig. 5:**
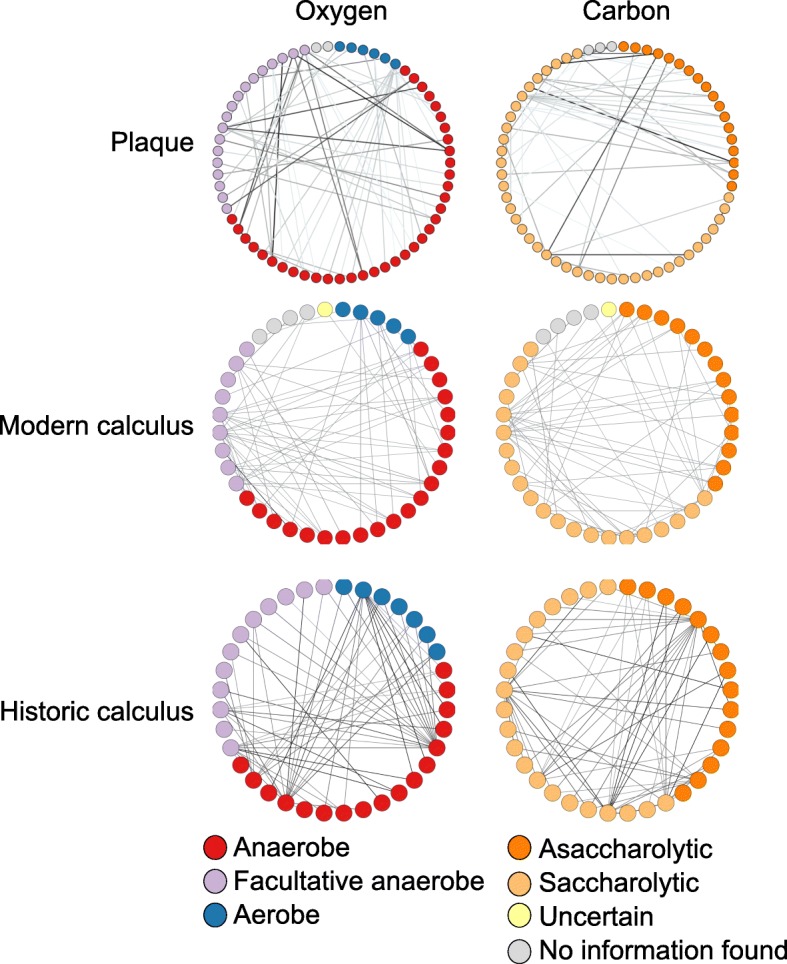
Microbial co-exclusion patterns reflect oxygen use and carbon source. Network graphs presenting species as nodes and co-exclusion between species as edges, where darker lines indicate stronger co-exclusions. Nodes are colored based on oxygen tolerance (left) and carbon source (right). Identical networks with labels indicating the bacterial species are presented in Additional file 1: Figure S10A,B. Historic calculus has more co-exclusions between species with different oxygen tolerance and carbon source than does plaque or modern calculus

To further investigate the differences in microbial profiles between plaque and calculus, we compared the patterns of species co-exclusion within each substrate using the program CoEx [27]. Species co-exclusion, or a negative correlation between the presence and abundance of two species, may indicate competition, antagonistic interactions, or different environmental preferences and may offer insights into the microbial community differences we observed between plaque and calculus. We visualized co-exclusion patterns in plaque, modern calculus, and historic calculus samples using network graphs (Fig. 5, Additional file 1: Figure S10A,B), with nodes representing species and edges representing co-exclusion, with stronger co-exclusions indicated by thicker, darker lines. Nodes were colored based on oxygen tolerance (aerobe, facultative anaerobe, anaerobe), use of sugars as a carbon source (saccharolytic, asaccharolytic), Gram stain (positive, negative), and phylum, to determine if co-exclusion was related to these characteristics.

No clear relationship was observed with Gram stain or phylum for plaque, modern calculus, or historic calculus (Additional file 1: Figure S10B). However, the majority of most strongly supported co-exclusions, those visualized in our graphs (Fig. 5), are between species with different oxygen tolerance (85% in plaque, 91% in modern calculus, 96% in historic calculus), different carbon source utilization (48% plaque, 51% modern calculus, 75% historic calculus), or both (43% plaque, 51% modern calculus, 73% historic calculus). The co-exclusion patterns between plaque and calculus are largely unique to the sample type (plaque, modern calculus, or historic calculus), with only 1.5% of all co-exclusions in modern calculus and 0.54% of all co-exclusion in historic calculus also reported in plaque, while these shared co-exclusions make up only 0.8% of all plaque co-exclusions (Additional file 2: Table S5). Historic and modern calculus samples have more shared co-exclusion patterns with each other than with plaque, with 19% of all co-exclusions in modern calculus and 6.8% of all co-exclusion in historic calculus reported in both, suggesting different environmental conditions in calculus compared to plaque.

### Microbial complexes in plaque and calculus

**Fig. 6:**
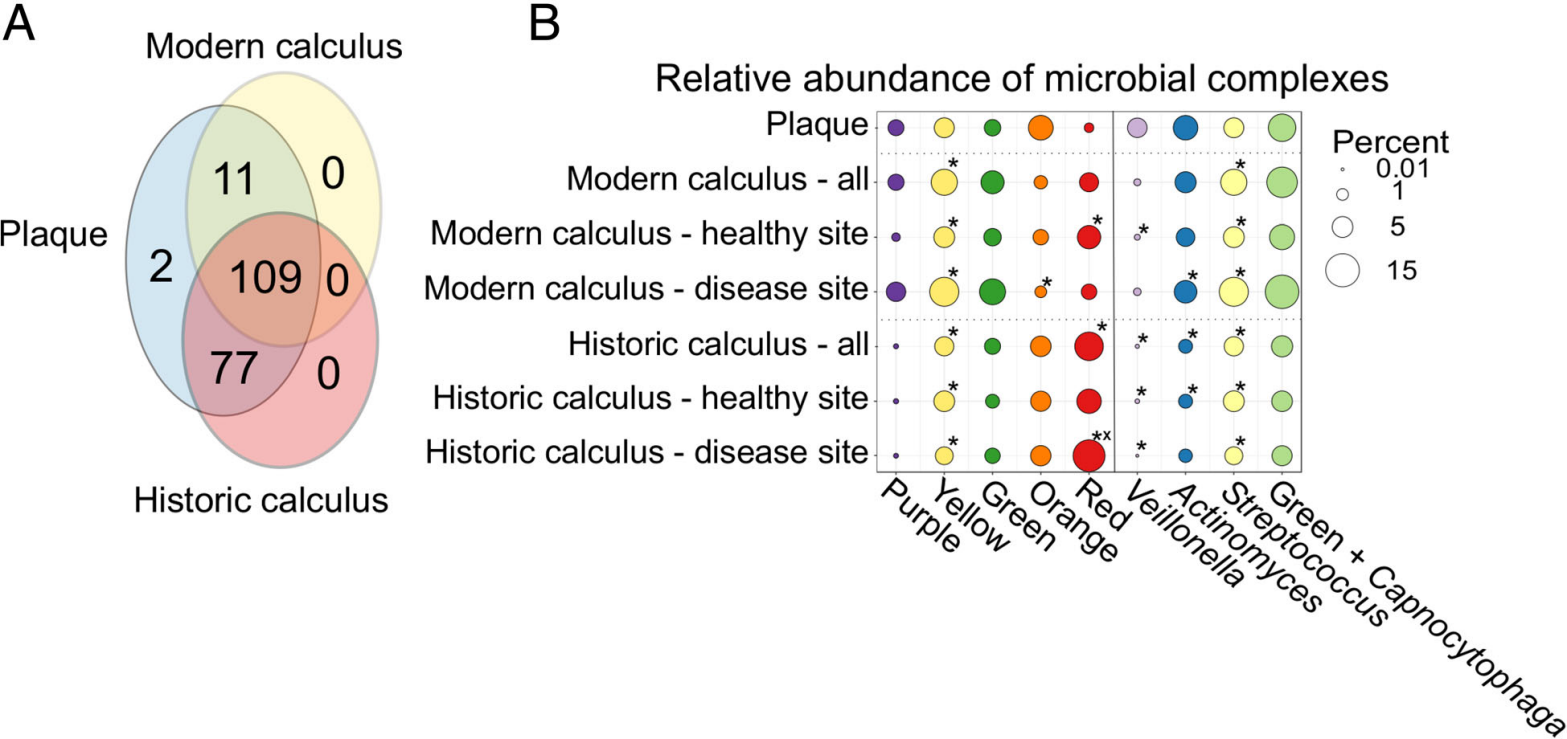
Microbial species differences between plaque, modern calculus, and historic calculus. **a** The majority of taxa detected are shared between plaque, modern calculus, and historic calculus. **b** Relative abundance of Socransky’s bacterial complexes in plaque and calculus, presented by age and health status. *p < 0.05 vs. modern plaque, ^x^*p* < 0.05 vs. modern healthy site calculus

The majority of species detected in plaque and calculus samples were shared between the groups, with 109 of 199 species detected in all three groups (Fig. 6a). The average number of species detected in historic calculus was less than in modern calculus or plaque (Additional file 2: Table S4), and the wide standard deviation in each group may be partially related to the sequencing depth (Additional file 1: Figure S11). Because the proportions of several species in our calculus samples deviated strongly from those in the plaque samples, we next investigated the differences in species profiles using Socransky’s microbial complexes scheme [17]. These complexes, named by color, consist of species from subgingival plaque that were significantly associated with each other by cluster analysis, and the clusters are associated with clinical periodontal parameters of health (purple, yellow, and green complexes) and disease (orange and red complexes). For each complex, we summed the proportion of all of the species that comprise it within the plaque, all modern calculus, and all historic calculus, as well as separating the calculus samples by disease status, presented as a bubble chart (Fig. 6b). Separately, we summed the proportion of all *Veillonella*, *Actinomyces*, *Streptococcus*, and *Capnocytophaga* species (Fig. 6b), as they are closely related to the species making up the purple, yellow, and green complexes, and assessed these groups in parallel.

The differences in the proportions of each complex as well as the species groups between plaque, modern calculus, and historic calculus indicate that modern calculus profiles are intermediate between plaque and historic calculus. Overall proportions of yellow, green, and orange complex species are similar across the three groups, while the purple complex is far lower in abundance in historic calculus than plaque, and the red complex is greater in both historic and modern calculus than in plaque. The proportions of the yellow complex and all *Streptococcus* species are significantly different in all calculus samples than modern plaque (*p* < 0.05), while the proportions of the red complex, *Veillonella* species, and *Actinomyces* species are significantly different between historic calculus and modern plaque (*p* < 0.05). The relative proportion of each species in each complex (Additional file 1: Figures S12, S13) demonstrates that the patterns of species abundance are different between plaque, modern calculus, and historic calculus, while there is very little difference in the proportions between healthy site and disease site historic calculus samples. Notably, the red complex species are much higher in calculus than plaque (Additional file 1: Figure S12), particularly *Tannerella forsythia*, a difference that we found that drives the classification of calculus from plaque (Additional file 1: Figure S4A,B), while disease site historic calculus has higher levels of red complex species than healthy site calculus, a pattern also seen in plaque of periodontal disease patients.

### Functional prediction in calculus is poorly predictive of health status

**Fig. 7:**
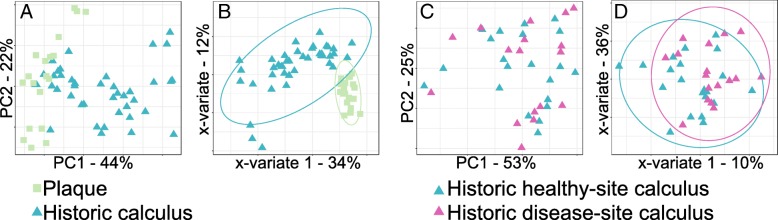
Potential metabolic functional profiles differ by sample type but not health status. **a** SEED metabolic functional category profiles separate plaque and historic calculus in a principal component analysis. **b** SEED profiles of plaque and historic calculus are sufficiently discriminatory to cluster samples by type in the sparse partial least squares-discriminant analysis (sPLS-DA). **c** Healthy and periodontal disease site historic calculus SEED profiles overlap and are not distinctly separated by PCA. **d** Historic healthy and disease site calculus SEED profiles overlap and are not sufficiently discriminatory for distinct clustering by sPLS-DA

Predicting metabolic functional capacity of a microbial community through gene content analysis is a way of inferring its potential activity in the absence of transcriptomics, proteomics, or metabolomics profiling. Differences in metabolic functions of microbial communities may indicate shifts in community activity associated with a changing environment, often linked to disturbed host physiology [28, 29]. Here, we compared the metabolic functional profiles of our historic calculus and modern plaque samples based on SEED subsystem classification to determine if the microbial communities in these two substrates were systematically enriched or depleted in any metabolic pathway categories (Additional file 2: Table S6). A PCA of the SEED profiles of plaque and historic calculus show some separation of the substrates (Fig. 7a, Additional file 1: Figure S14A), but group separation was not significant and no SEED categories were significantly differentially abundant between them. The sPLS-DA plot indicates that the sample types are discriminative, but the calculus samples are more variable than the plaque samples (Fig. 7b), which is reflected in the BER between the sample types (Additional file 2: Table S3). The SEED categories Iron Acquisition and Potassium Metabolism, which are associated with healthy site plaque in our results, and Protein Metabolism and Sulfur Metabolism, which are associated with healthy site calculus in our results (Additional file 1: Figure S14B), are associated with periodontal disease through clinical and laboratory studies [28, 30]. This was unexpected, given that we have demonstrated that the microbial profile of calculus contains microbial signatures characteristic of periodontal disease, and we expected the SEED profiles to similarly reflect signatures of disease in calculus but not plaque.

Because previously published transcriptomic analyses of plaque from healthy and periodontitis sites indicate differential expression of specific metabolic pathways in each condition, even when microbial profiles vary between samples of the same condition [31, 32], we further assessed if potential metabolic profiles differ between our historic calculus samples from periodontitis-affected and healthy sites. However, a PCA of SEED categories between healthy site and disease site calculus showed no separation of these groups (Fig. 7c). Furthermore, sPLS-DA was not able to sufficiently distinguish between the sample types for distinct classification (Fig. 7d), the BER for sample classification was high for both sample types (Additional file 2: Table S3), and only five SEED categories were informative enough for classification (Additional file 1: Figure S14C). Sulfur metabolism and potassium metabolism in our data contribute to the classification of healthy site samples but in plaque are associated with periodontitis [28, 33]. The differences in SEED profiles between plaque and calculus and our inability to distinguish between SEED profiles of healthy and disease site calculus results are consistent with the taxonomic profile differences we observed. Both results support that calculus samples contain species and gene content profiles distinct from plaque, but differences in disease and healthy site profiles are not evident at the taxonomic/genetic level. This is reinforced by the consistent relative abundance of the top 15 most abundant SEED categories across all historic calculus samples despite health status (Additional file 1: Figure S15).

### Proteomic profiles of historic healthy site calculus

**Fig. 8:**
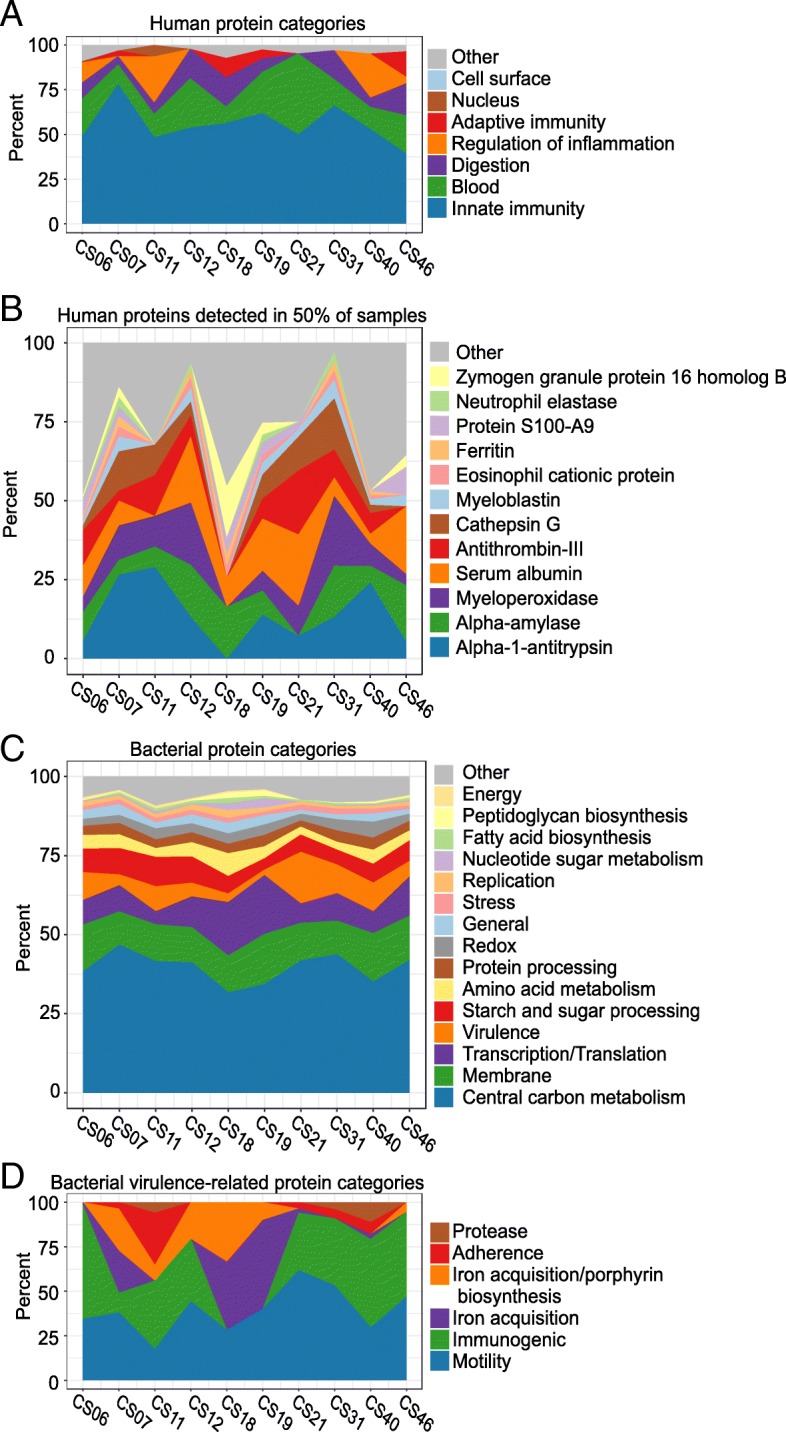
Historic calculus protein profiles reflect host homeostasis. Filled area charts presenting relative abundance in each sample of **a** host protein categories, **b** human proteins detected in at least 50% of the samples, **c** bacterial protein categories, and **d** bacterial virulence protein categories

In contrast to genetic data, proteins dynamically reflect biofilm and host processes and may be more useful for understanding active cellular processes in the oral environment. Therefore, to gain insight into the protein profile of our historic calculus samples, we performed shotgun proteomics on a subset of healthy site historic dental calculus samples (*n* = 10) and manually annotated the functional properties of all proteins. This dataset was previously assessed for the presence of dietary proteins, but none were identified [6]. We found that the majority of proteins (94.9%) were derived from bacteria, and the remaining 5.9% were from the human host (Additional file 2: Table S7). Consistent with previous studies [2, 34], immune response proteins make up nearly 50% of all human proteins identified in each calculus sample (Fig. 8a), with innate response-related molecules (e.g., myeloperoxidase, cathepsin G) more prevalent and abundant than immunoglobulins of the adaptive response. Many of the identified blood-associated proteins (e.g., antithrombin-III) are known to be involved in clotting and likely come from the gingival crevicular fluid. Alpha-amylase, the sole protein in the digestion category, was found to be the third most abundant protein after the innate immune protein alpha-1-antitrypsin and the blood protein serum albumin; however, none of the human proteins was detected in all 10 samples (Fig. 8b).

Bacterial protein categories were represented more evenly across the samples than the human protein categories (Fig. 8c). Those involved in central carbon metabolism dominate the bacterial protein profile, while membrane proteins are the second most abundant category, accounting for approximately 12% of the bacterial proteins in each sample. When including membrane-associated proteins involved in virulence, such as the *Porphyromonas gingivalis* gingipains and fimbrial proteins, the membrane-bound proteins make up 16% of all bacterial proteins. Proteins related to virulence were the fourth most abundant category, and we looked at these in more detail to understand why we observed a robust immune response signal in samples from apparently healthy teeth. Flagellar proteins were abundant in nine samples, as were the immunogenic *Tannerella forsythia* surface layer proteins A and B (Fig. 8d). Samples with gingipains, including either or both of arginine gingipain Gingipain R1, a highly antigenic *Porphyromonas gingivalis* protease [35], or lysine gingipain, also had *P. gingivalis*-specific major and minor fimbriae and hemagglutinin, which are involved in adherence to host epithelial cells.

### Correlations between taxonomic, proteomic, and metabolomic profiles

**Fig. 9:**
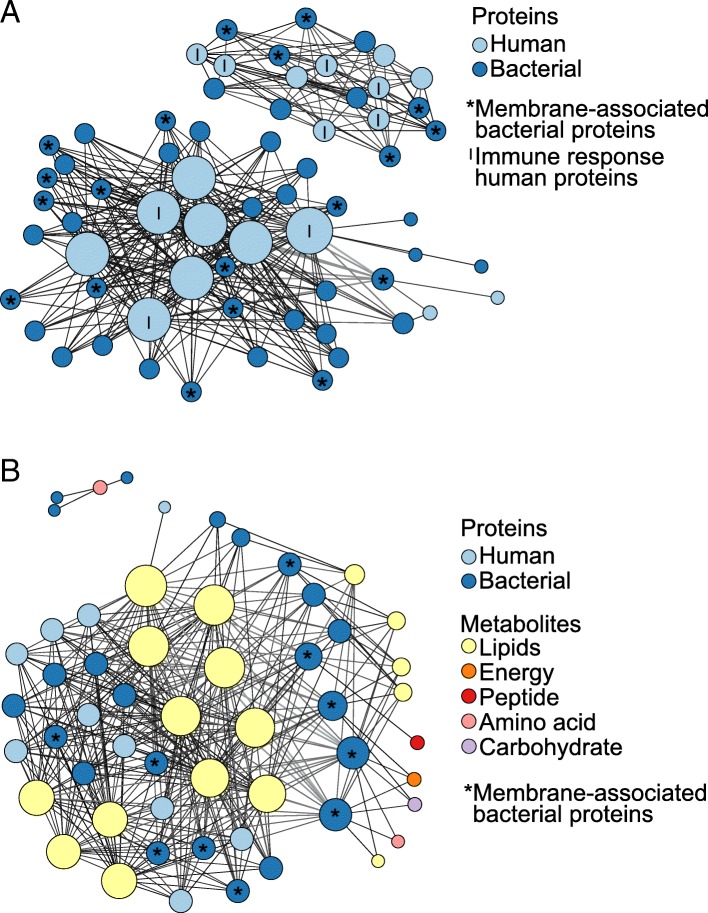
Canonical correlations between historic sample proteins and metabolites. The strongest canonical correlations in historic calculus are presented as network graphs where the nodes are proteins/metabolites and edges represent canonical correlations, with darker, thinner lines lower values and thicker, lighter lines higher values. Nodes are sized by the number of edges they have and are colored based protein on metabolite category. **a** Bacterial and human protein correlations ≥ 0.9. **b** All protein and metabolite correlations ≥ 0.9. Graphs with node names are included in Additional file 1: Figures S18-S19

Previous research has shown that a wide range of small molecule metabolites preserve in dental calculus, possibly enabling integrative multi-omic studies of historic dental calculus. The metabolite profiles of a subset of our historic dental calculus samples were previously studied in Velsko et al. [5], and we incorporated these results into this study by testing for correlations between our genomic, proteomic, and metabolomic data in the samples for which we have overlapping datasets. We performed regularized canonical correlation analysis (rCCA) between the taxonomic and protein profiles (*n* = 9), taxonomic and metabolomic profiles (*n* = 11), proteomic and metabolomic profiles (*n* = 7), and bacterial and human protein profiles (*n* = 10) and visualized the strongest correlations (~ 350 edges with the highest correlation values) with network graphs (Fig. 9, Additional file 1: Figures S16-S19). The nodes indicate the species/proteins/metabolites, and the edges are the canonical correlation value, where smaller darker lines are lower values and the thicker, lighter lines are higher values.

All of the ~ 350 strongest correlations between taxonomy and metabolites, proteins and metabolites, and bacterial and human proteins were positive, and all but four of the strongest correlations between taxonomy and proteins were positive. The biological basis for the correlations between taxonomy and proteins or metabolites is unclear, but the protein and metabolite correlations appear to be related to immunological interactions and biomolecule preservation patterns. For example, in examining the strongest bacterial-human protein correlations (Fig. 9a), just over half of the bacterial proteins (19/36) are membrane-associated and half of the human proteins (9/18) are involved in the immune response, which may be because surface proteins are exposed for immune system interactions. In the strongest metabolite-protein correlations, the majority of metabolites in the network are lipids, which is consistent with the observation that lipids are the most abundant metabolite class in historic dental calculus [5]. Of the 33 proteins in the network graph, 23 are from bacteria, and 10 of these (43%) are membrane-associated. This is the highest proportion of all bacterial protein classes represented in the strongest correlations. Nine of the 17 lipid metabolites are known components of bacterial membrane lipids, and therefore, membrane-associated proteins may be stably preserved by the relatively chemically inert lipid membranes.

## Discussion

We have demonstrated that microbial profiles of historic and modern calculus are highly similar to each other, albeit with several notable differences, and both are distinct from dental plaque. The systematic microbial profile differences observed between plaque and calculus complicate our ability to infer microbial population shifts related to dietary, social, medical, or geographic changes in populations, and studies should take care when comparing microbial profiles between the two substrates, as this can lead to inappropriate data interpretation [36]. For example, microbial species profiles between dental plaque and calculus can be strikingly different, such that substantial community shifts appear to have occurred over the last several hundred years, concurrent with industrialization in Western societies [1]. To overcome this challenge, we strongly recommend using reference datasets derived from modern dental calculus, such as the one provided in this study, rather than dental plaque, when performing investigations of oral microbiome ecological and evolutionary change.

Our results suggest that species strongly associated with periodontal disease today may in part be more frequently detected and more abundant in archeological calculus simply because past oral biofilms represent more mature microbial communities. The modern calculus samples examined in this study contain high proportions of “early colonizer” *Veillonella* and *Capnocytophaga* species similar to those in plaque, yet the historic calculus samples have very little *Veillonella* and lower proportions of *Capnocytophaga*, while proportions of “early colonizer” *Actinomyces* in modern calculus appear intermediate between plaque and historic calculus. Similarly, the proportions of “orange complex” and “red complex” species in modern calculus are intermediate between historic calculus, in which they are high, and plaque, in which they are low. Notably, the “red complex” species *P. gingivalis*, *T. denticola*, and *T. forsythia* are found in substantially higher proportions in dental calculus compared to plaque, whether the calculus is modern or historic and from a healthy site or a disease-affected site. The presence/absence and abundance in archeological calculus of *P. gingivalis*, *T. forsythia*, and *S. mutans* have been used as proxies for the presence of oral disease and biofilm pathogenicity [1, 2], yet this is unlikely to be reliable.

In contrast to red complex bacteria, *S. mutans*, a species strongly associated with dental caries, is often difficult to detect in historic calculus, even from samples collected from teeth with severe carious lesions, and we did not detect it in our samples (Additional file 2: Table S2). This may be related to *S. mutans* biofilm physiology, which is now being clarified with next-generation sequencing [37–39]. Although this species grows prolifically in a laboratory setting and can thrive under a range of environmental conditions [40], it is less common in biofilms from early compared to advanced caries lesions [37–39]. If *S. mutans* grows best in deep, advanced lesions that have reached the dentin or pulp, it may be infrequently detected in calculus from the tooth surface and subsequently lost during decomposition of the soft tissues of the pulp. Additionally, *S. mutans* produces acids that demineralize the tooth enamel and dentin; these acids will also inhibit biofilm mineralization, such that biofilms with abundant acid-producing *S. mutans* may not calcify (and therefore not preserve) to the extent of biofilms without *S. mutans*.

Examining the potential metabolic functional profile of the microbial gut [41] and subgingival plaque [42] communities has revealed the differences in enriched gene classes between health- and disease-associated communities. Such gene content differences potentially explain which bacterial metabolic pathways are altered concomitant with host disease, providing possible explanations for disease development and progression. Dental calculus microbial gene content does not appear to be similarly reflective of health status, as healthy and periodontitis site calculus samples had highly congruent SEED category profiles. Rather, for dental calculus, gene expression measured by proteomics and/or metabolomics may be a more accurate and reliable method of distinguishing healthy and disease site biofilm metabolic activity [5, 29, 31, 32, 43].

In this study, we were unable to explore protein-level differences in healthy site and disease site historic calculus, however, as all ten of the calculus samples we used for proteomic profiling came from teeth with no evidence of periodontal disease. These samples still aid our understanding of health-associated biofilm environments, which is crucial to differentiating health from disease states, and for understanding disease development and progression. A robust immune response characterizes periodontal disease, and an innate inflammatory response is conventionally associated with early disease, while an adaptive response is associated with advanced disease [44]. Gingivitis, an early stage soft tissue inflammatory condition, is likely to have affected many, if not all, of the teeth from which healthy site calculus was collected, and we saw a protein profile that reflected an early inflammatory response. Innate immune response proteins dominated the host response protein profile, and as has been reported previously in calculus [2], many of these proteins are produced by neutrophils. Several proteins involved in regulating immune responses were also detected in the calculus samples, including leukocyte elastase inhibitor and serpins B3, B6, and B10 and may indicate appropriate control of inflammation at healthy tooth sites.

There are correlations between detection of species-specific proteins and the relative abundance of a given species by DNA sequencing. Samples in which *P. gingivalis*-specific proteins or *T. forsythia*-specific proteins were detected have a higher relative abundance of those species as determined by genetic sequencing than the samples in which no *P. gingivalis*- or *T. forsythia*-specific proteins were detected. This may indicate a minimum relative abundance of a species for its proteins to be reliably detected in shotgun proteomics, as well as suggesting that the activity of these species was not disproportionate with their relative abundance. It will be of interest in future studies to determine if the proportion of proteins detected (i.e., the apparent activity level) from periodontal disease-associated species such as *P. gingivalis* or *T. forsythia* is disproportionately higher in samples collected from teeth with evidence of periodontal disease.

## Conclusions

Ancient dental calculus is an exceptional substrate that allows the direct investigation of oral microbiome evolution, host immune responses, and dietary change through time. Clinical studies of the oral microbiome typically focus on dental plaque, the living biofilm from which calculus forms, rather than calculus, and to date, many studies have treated the two substrates interchangeably. We have shown, however, that while these two substrates share considerable taxonomic overlap, they are microbiologically distinct from each other. Modern oral hygiene practices that disrupt natural oral biofilm development and maturation may be responsible for the major differences we observed, and thus caution should be exercised when quantitatively comparing the two substrates. To more accurately study how the oral microbiome has evolved through time in relation to human cultural and dietary changes, we recommend using modern calculus rather than plaque biofilms as a reference. In addition, studies of ancient dental calculus that incorporate metagenomic, metaproteomic, and/or metabolomic data have the potential to reveal substantial insight into the oral biofilm and host physiology, approximating the detailed profiles that can be generated on modern microbiome samples. Such multi-omic studies would provide an authentic historic example of human-microbiome co-evolution and could offer unique insights into health and disease processes.

## Materials and methods

### Historic and modern calculus sample collection

Fresh dental calculus samples (*n* = 10) were obtained from a private dental office in Jaén, Spain, during routine dental cleaning. Calculus was collected by dental professionals using a dental scaler following standard calculus removal procedures. The collection site (healthy or diseased) was selected arbitrarily by the dentist. All samples were obtained under informed consent, and this research was reviewed and approved by the University of Oklahoma Health Sciences Center Institutional Review Board (IRB #4543). Historic dental calculus (ca. 1770–1855) (*n* = 48) was collected from the skeletons in the Radcliffe Infirmary Burial Ground collection [45], housed at Oxford Archaeology in Oxford, UK. All of the skeletons were excavated from earth-cut graves and had either been contained within wooden coffins, subsequently decomposed, or had been buried in shrouds without coffins, and the skeletons are not personally identifiable.

The oral health of each skeleton was recorded with reference to the presence or absence of caries and periodontal disease following previous guidelines [46, 47]. Briefly, periodontal disease refers to the inflammatory loss of the alveolar bone and was recorded following Ogden [47]. This method involves a 4-point scoring system (1–4 in which 1 is “no disease” and 4 is “severe periodontitis”) and controls for confusion with compensatory eruption by focusing on the morphology of the alveolar margin, rather than the amount of tooth root exposed (ibid).

The sex and approximate age at death for each skeleton were estimated following established osteological criteria [48–53] and are presented in Additional file 2: Table S1. Genetic sex was assessed through high-throughput sequencing (HTS) of DNA extracted from the calculus fragments (see below) following previously described methods [54–56]. Genetic sex determinations were consistent with those made using osteological approaches for most of the samples (Additional file 2: Table S1). In the four cases of conflicting assignments between genetic and osteological analyses, the osteological assessment was noted as uncertain, and genetic sex was used for all subsequent analyses. In several instances, insufficient human DNA was recovered for genetic sex determination, and the osteologically determined sex was used for subsequent analyses.

Historic calculus samples were collected on site as follows: surfaces of the teeth and calculus were cleaned with Kimwipes moistened with 5% NaOCl followed by water prior to sampling to remove traces of burial soil. The jaws, or individual teeth if they were loose, were photographed, and sampling was performed wearing gloves and a mask over the nose and mouth. Calculus was collected from each individual using a dental scaler onto a clean piece of aluminum foil and then transferred into a sterile 1.5 mL tube. Between individuals, the scaler was cleaned using 5% NaOCl and rinsed with ultra-pure water. The samples were transferred to the Research Laboratory for Archaeology and the History of Art at the University of Oxford for DNA and protein extraction. Metadata collected for each sample are presented in Additional file 2: Table S1 and include the following: estimated age and sex (see below), mandible/maxilla, tooth, tooth surface (buccal/lingual), deposit location on tooth (crown, root, cemento-enamel junction), deposit density (thick or thin), deposit spread (contained ring or “blanket” over the tooth surface), single tooth sample or pooled sample, presence of caries and/or periodontitis on the sampled tooth, and presence of caries and/or periodontitis on non-sampled teeth (whole mouth caries or whole mouth periodontal disease)

### DNA extraction

All Radcliffe calculus sample DNA extraction and library building were performed in the PalaeoBARN dedicated ancient DNA laboratory at the University of Oxford Research Laboratory for Archaeology and the History of Art. The Radcliffe calculus samples were sectioned, and pieces of approximately the same size as a previously weighed 40 mg piece of calculus were selected for DNA extraction. The modern calculus samples were extracted using the DNeasy PowerSoil kit (QIAGEN) as used in the Human Microbiome Project extractions. For details, see Additional file 1: Supplemental Methods.

### DNA library construction and high-throughput sequencing

Shotgun Illumina libraries of the Radcliffe calculus samples were constructed following previously described methods [57] with the AccuPrime PFX (Invitrogen) and KAPA HiFi Uracil+ (Roche) polymerases. Libraries were dual-indexed with one internal 6 bp index and one external 6 bp index. The proof-reading capability of the AccuPrimePFX enzyme impairs PCR amplification from templates with DNA damage (cytosine deamination), while the KAPA HiFi Uracil+ enzyme does not have this capability. Four samples failed to build successful libraries with the AccuPrime PFX polymerase, which was later determined to be due to an error with the internal index on those samples, and these libraries are not included in the downstream microbial profiling analyses. The four samples were successfully built into libraries with the KAPA HiFi Uracil+ polymerase using different internal indices and were included in damage pattern assessment analysis. For details, see Additional file 1: Supplemental Methods.

### DNA sequence processing

Prior to analysis, reads were de-multiplexed, quality-checked, and trimmed of adapters using AdapterRemoval v.1 (Lindgreen 2012) with the following non-default parameters: --maxns 0, --trimns, --trimqualities --minquality 30, --minlength 25, --collapse, and --minalignmentlength 10. The AccuPrimePFX enzyme-generated reads were used for all subsequent analyses, while the KAPA HiFi Uracil+ enzyme-generated reads were used for DNA damage pattern analysis with mapDamage2 [58, 59].

For the 10 modern calculus samples, sequencing reads were processed using the EAGER pipeline v1.92.55 [60]. In brief, reads were quality checked with FastQC (https://www.bioinformatics.babraham.ac.uk/projects/fastqc/). Forward and reverse reads were trimmed and merged using AdapterRemoval2 [61], with the following parameters: --trimns –trimqualities –minlength 30 –minquality 20 –minadapteroverlap 1. Merged reads were then mapped to the human reference genome (HG19, http://hgdownload.cse.ucsc.edu/downloads.html#human) using bwa aln [62] v0.7.12, with -n 0.01 -l 32. Samtools v1.3 [63] was then used to convert to bam format, generate mapping statistics, and extract unmapped reads using the view function’s -f4. The samtools fastq function was then used to convert the unmapped reads back to fastq format for downstream taxonomic profiling.

Ten supragingival plaque samples and ten subgingival plaque samples from the Human Microbiome Project (HMP) cohort were downloaded from the HMP web server. Only the pair1/pair2 files were processed, and singletons were excluded. The samples were quality-checked and trimmed of adapters using AdapterRemoval with the same settings as for the historic calculus samples above.

### Genetic assessment of historic calculus sample preservation

Preservation was assessed by damage pattern characterization and microbial source profiling. Damage patterns were assessed using mapDamage2 [59] on the Radcliffe calculus libraries that were generated with the KAPAHiFi Uracil+ polymerase. For mapDamage, all calculus sample reads were mapped to the *Tannerella forsythia* 92A2 genome (assembly GCA_000238215.1) using bwa aln with the flags -l 1024 -n 0.03 [64], and duplicates were removed from the alignment using samtools [63]. The mapped, non-duplicate reads were assessed for cytosine to thymine transitions and breakpoints coinciding with depurination in mapDamage2, run with default parameters. The Bayesian analysis-based program SourceTracker [65] was used to estimate the source composition of the AccuPrimePFX enzyme-generated library microbial communities as assessed from 16S rRNA gene reads processed in QIIME, exactly as described in [5]. Human reads were identified as described in [5].

### Genetic microbial taxonomic profiling

The microbial profile of the Radcliffe historic calculus, Human Microbiome Project supra- and subgingival plaque samples, and modern calculus samples were determined by MetaPhlAn2 [66, 67], a profiler selected based on Velsko et al. [23]. All analysis-ready reads from the Radcliffe historic and Spanish modern datasets were profiled using MetaPhlAn2 in default parameters, while the HMP samples were subset to 10,000,000 analysis-ready reads using seqtk sample (https://github.com/lh3/seqtk) before profiling, to keep the number of input reads within the range of the historic calculus samples. The species-level assignments were extracted from the MetaPhlAn2 output tables (Additional file 2: Table S2) and used for all further analyses. Bubble charts of the relative abundance of species or species complexes (defined by Socransky et. al [17]) were generated with ggplot2 (https://ggplot2.tidyverse.org/) in R.

### Principal component analysis

Principal component analysis (PCA) was performed using the R package mixOmics [68]. The species-level relative abundance tables generated by MetaPhlAn2 were filtered to include only species present at > 0.02% relative abundance and used as an input. Within the mixOmics package, the tables were offset by + 1 to allow the use of the centered-log ratio (CLR) transformation, followed by total sum scaling (TSS) normalization. Principal component analysis was run with 10 components, CLR data transformation (Gloor), and data centering. Scree plots were visually inspected to assess the variation explained by each component. Plots of each PCA were generated with mixOmics. Group differences were tested on the distance matrices using adonis in the vegan R package, with 999 permutations, and *p* < 0.05 considered significant. No groups tested were significantly different. Analyses were also performed without total sum scaling normalization and all results were identical—the proportion of variance explained by each of the components was identical between TSS-normalized and non-TSS-normalized datasets.

### Assessment of differentially abundant taxa

Differential abundance of species between selected sample groups was determined using the program Statistical Analysis of Metagenomic Profiles (STAMP) [69, 70]. Tables filtered to include only species at > 0.02% relative abundance were analyzed by White’s non-parametric two-sided *t* test with bootstrapping to determine the difference between proportion (DP) with cutoff 95% and Storey’s FDR. Corrected *p* values (*q* values) of ≤ 0.05 together with an effect size ≥ 1 were considered significant. To determine if the proportion of species complexes (Fig. 6b) were different between plaque and calculus samples, an ANOVA with multiple comparisons and uncorrected Dunn’s test was performed, and *p* < 0.05 was considered significant.

### Sparse partial least squares-discriminant analysis

Sparse partial least squares-discriminant analysis (sPLS-DA) was performed for each metadata category of interest (sample source, sample health status) with the species tables using the R package mixOmics [68], following the example Case Study sPLSDA: SRBCT available on the mixOmics website (http://mixomics.org/case-studies/splsda-srbct/). sPLS-DA is a method of data analysis that starts with the knowledge of the classification of each sample. It then looks for the data (in this case species) that maximize the differences between the sample categories, i.e., the data that discriminate the sample categories from each other. sPLS-DA is used for sample classification, unlike PCA, which is used for data exploration, and can be applied to biomarker discovery, and further for classification of samples from unknown sources in the future.

The input tables and data pre-processing were identical to that in the PCA section described above (offset by + 1, TSS). In brief, a PLS-DA was run with 5 components and CLR transformation to assess the number of components to be included in the sPLS-DA. The sPLS-DA was tuned using the tune.splsda function to determine the optimal number of variables to select, then the sPLS-DA was run with the selected number of variables. Plots of each sPLS-DA were generated in mixOmics with plotVar. The top 20 species or genera contributing to the loadings of components 1 and 2 were plotted with plotLoadings function. These are the taxa that contribute the most to the separation of the two groups being compared. The balanced error rate (BER) of group classification for each sPLS-DA (Additional file 2: Table S3) was calculated with the tune.splsda function in mixOmics. The BER is a measure of performance calculated from sensitivity and specificity, and lower numbers indicate more accurate classification.

### Assessment of microbial co-exclusion patterns

We assessed microbial species co-exclusion patterns in our sample groups using the program CoEx [27]. Relative abundance tables filtered to include only species/genera at > 0.02% were used as input, with the following parameters: -n 50 -f 100 -t 10000 -p 0.05 -z 0.6 -norm. For graphical assessment of co-exclusion patterns, the data were visualized as network graphs with the species/genera as nodes and the co-exclusion value as edges, using Gephi [71]. To make the network graphs readable, only the top 60 species-level co-exclusion interactions and top 40 genus-level co-exclusion interactions that met the following criteria were plotted: *p* < 0.05, co-exclusion value < 0.1, false discovery rate for the given *p* value < 1%. The nodes were colored by Gram stain (positive, negative, unknown), oxygen tolerance (aerobe, facultative anaerobe, anaerobe, unknown), utilization of sugars as a carbon source (saccharolytic, asaccharolytic), and by phylum, to look for characteristics that play a role in co-exclusion patterns.

### Gene functional categorization with SEED

To generate SEED protein category classification [72] for the Radcliffe calculus and HMP plaque samples, the analysis-ready reads for both were profiled using MALT-X [36] in default mode with a custom database of NCBI RefSeq genomes of bacteria, viruses, archaea, and plasmids from Velsko et al. [23]. SEED categorization was added in MEGAN v. 6.10 [73] using the acc2seed-May2015XX mapping file. SEED tables were exported from MEGAN (Additional file 2: Table S6), and categories that were present at less than 0.02% abundance were removed for analysis, for consistency with taxonomic profiling. The highest level of SEED categorization was used for all analyses, which included PCA, differential abundance assessment, and sPLS-DA as described above for analysis of taxonomic profiles.

### Proteomics

The historic Radcliffe Infirmary proteomic data presented here are from Hendy et al. [6], and sample processing is described therein. The 10 historic samples were selected based on having sufficient material for protein extraction after pieces were used for DNA extraction and include both males and females. All dental calculus samples were obtained from teeth without evidence of periodontal disease. Peptides were extracted using the GASP method, and shotgun sequenced at the Oxford Target Discovery Institute. Mass spectrometry data were analyzed according to Jeong et al. [74] with minor changes. Briefly, spectral data generated via MS/MS were converted to Mascot generic format using the application MSConvert [75]. The resulting files were searched for peptide spectral matches using Mascot (Matrix ScienceTM, version 2.6) against SwissProt and a database made up of 463 annotated bacterial genomes from the Human Oral Microbiome Database [76] (downloaded 2017). Each database also contained a reverse decoy of every sequence which was used in the downstream analysis to calculate the false discovery rate (FDR). Duplicate peptides were removed, and only the proteins supported by a minimum of two peptides, each with an *E* value ≤ 0.01, were used to calculate FDR at both the protein and peptide level across the dataset [74].

For data analysis, all protein names were manually checked and variations on the same protein were made consistent (i.e., variations of “GroEL” such as “chaperone GroEL” were re-named “GroEL”). Proteins were manually assigned to a broad-level category based on KEGG orthology when available, and based on functional descriptions when no KEGG entry was found, to examine active cellular processes (Additional file 2: Table S7). Graphs showing the relative abundance of protein categories were generated in R.

### Metabolomics

The historic Radcliffe Infirmary and modern metabolite data presented here are from Velsko et al. [5]. All metabolites detected in at least one historic calculus sample were included in regularized canonical correlation analyses described below.

### Regularized canonical correlation analysis

We used the mixOmics R package [68] to perform regularized canonical correlation analyses on the taxonomic, proteomic, and metabolomic datasets. The tables used for analysis were filtered using the same criteria as above: only species present at greater than 0.02% abundance, only proteins detected at least twice by independent peptides, and metabolites detected in at least one historic sample. The input tables were offset by + 1, and the rCCA was run using the shrinkage method to estimate penalization parameters. The matrix of canonical correlation values was exported, and the strongest correlations were visualized as network graphs with Gephi as above for co-exclusion patterns. Nodes are species, proteins, or metabolites, and edges are canonical correlation values. Cutoff correlation values for network graph visualization were selected as follows because these were round numbers that left similar numbers of edges (343–368 edges): 0.9 for proteins-metabolites and bacterial-human proteins, 0.82 for species-proteins, and 0.75 for species-metabolites.

## Additional files

Additional file 1: Supplementary Materials and Methods and Supplementary Figures. (PDF 8386 kb)

Additional file 2: Supplemental Tables S1-S7. (XLSX 313 kb)
