# Supplementary material for: Microbial differences between dental plaque and historic dental calculus are related to oral biofilm maturation stage
Source: Microbiome. 2019 Jul 6;7:102. doi: 10.1186/s40168-019-0717-3 (PMC6612086; doi:10.1186/s40168-019-0717-3)
Supplement: Supplementary file 1 — Supplementary Materials and Methods and Supplementary Figures. (PDF 8386 kb) [file 40168_2019_717_MOESM1_ESM.pdf]

Supplemental Materials for
"Microbial differences between dental plaque and historic dental calculus are related to oral biofilm maturation stage"

1. **Supplementary Materials and Methods** 2
DNA Extraction – Historic calculus 2
DNA Extraction – Modern calculus 2
DNA library construction and high-throughput sequencing – *Historic calculus* 3
DNA library construction and high-throughput sequencing – *Modern calculus*. 4
2. **Supplementary Figures S1-S19** 5-24

1. Supplementary Materials and Methods

DNA extraction

Historic calculus

Each piece of calculus was put into a new 1.5 mL Eppendorf tube and crushed with an individual, sterile micropestle. To remove loosely bound contaminants, 1.5 mL of 0.5M EDTA was added to each tube, and the tubes were rotated at room temperature for 15 minutes, the calculus pelleted at 16000 g x 3 min, and the EDTA wash removed. A fresh 1.5 mL 0.5M EDTA was added to the tube and the pellet resuspended. Tubes were rotated for 72 hours at room temperature in the dark to decalcify. Samples were then centrifuged at 16000 g x 3 min, and 900 uL supernatant removed to a new tube. 90 uL proteinase K was then added to the supernatant, the tube gently vortexed, and rotated overnight at room temperature in the dark. The historic samples had proteinase K added after decalcification in order to allow for separate downstream proteomic analysis of the cellular debris pellet.

DNA was then extracted from the supernatant using a modified QIAGEN MinElute. In brief, the supernatant was transferred to a Zymo reservoir attached to a MinElute tube and diluted with 13 mL buffer PB, and gently mixed by pipetting. The mixture was centrifuged at 1500 g for 4 min, rotated 90°, centrifuged an additional 2 min at 1500 g, and the MinElute column transferred to a clean 2 mL tube. The MinElute was spun at 4000 g x 2 min, washed twice by adding 750 mL buffer PE and spinning at 800 g for 1 min. The MinElute column was transferred to a fresh 2 mL tube and DNA was eluted twice with the addition of 30 uL buffer EB directly to the MinElute membrane, followed by a 5 minute benchtop incubation, and then centrifugation at 200 g for 1 min. Approximately 50 uL of eluted DNA were collected and and stored at -80$_{o}$C to be built into libraries. One sample did not have sufficient calculus for analysis and yielded no DNA, as determined by Qubit. A blank extraction control was processed simultaneously with the calculus samples for each extraction batch.

Modern calculus

All of the work on modern calculus samples was performed in the lab facilities of the Max Planck Institute for the Science of Human History in Jena, Germany. Every batch of each protocol includes a blank control which were also sequenced. Each calculus sample (2-10mg) was washed with 1ml of 0.5M pH 8.0EDTA (Life Technologies) for 15min, while rotating at room temperature. The samples were then centrifuged for 1min at 16000 g and the supernatant stored at -20°C. Calculus was powdered with a clean metal pestle, and an extraction solution of 250uL of 0.5M pH 8.0 EDTA (Life Technologies) and 12.5uL 10mg/ml Proteinase K were added, and then incubated for 24-48h at room temperature on a rotator in the dark until the inorganic fraction was dissolved.

For the following steps the Dneasy Power Soil Kit (Qiagen) was used. The supernatants were transferred into Power Bead Tubes and gently vortexed. After adding 60uL C1-Solution, the tubes were inverted 5 times and secured on a flat-bed vortex pad. The samples were then vortexed at maximum speed for 10min. After centrifuging the samples for 30sec at 10000 g, the supernatants were transferred into a fresh collection tubes and mixed by vortexing with 200uL C3-Solution. After an incubation at 4°C for 5min the samples were centrifuged for 1min at 10000 g, and each supernatant was homogenised by vortexing for 5sec with 1.2ml C4-solution. 675uL of each supernatant was loaded on a Spin Filter, centrifuged for 1 min at 10,000xg and the flow-through discarded. This step was repeated until the solution was completely loaded. After loading 500uL of C5-Solution, the samples were centrifuged again (1min 10000 g), the flow-through were discarded, followed by a dry-spin step (1min 10000 g). The Spin Filter was placed into a fresh collection tube, and the DNA eluted with 100uL C6-

Solution, incubating for 1min on the 3 membrane, followed by a 30sec at 10,000xg centrifugation. The DNA extracts were then stored at -20°C.

The DNA was sheared using a M220 Focused-ultrasonicator (Covaris). For each extract 250 ng of DNA was mixed with AFA-Grade Water (Covaris) and set up for the DNA_0200bp_050_ul_Screw_Cap_microTUBE program as supplied by the manufacturer. The resulting sheared fragments had lengths of between 200 and 240bp, as measured by a 4200 TapeStation NucleicAcid System on the D1000 Kit (Agilent).

For each sample 100 ng of the sheared DNA was used to build a Illumina NGS library. The blunt ends of the DNA were repaired with 0.4 U T4 DNA Polymerase and 0.024 U T4 Polynucleotide Kinase (New England Biolabs), including NEB Buffer 2 (New England Biolabs), 1mM ATP (New England Biolabs), 0.8mg/ml BSA(New England Biolabs) 0.25mM dNTP's (Thermo Scientific), and balanced with water. The reactions were incubated for 15min at 15°C followed by 15min for 25°C. Afterwards the DNA was cleaned by MinElute Purification (Qiagen) with the manufacturers protocol, eluted in 20uL EB-Buffer (Qiagen) containing 0.05% Tween 20 (Sigma-Aldrich). The adapters were ligated with the Quick Ligation Kit (New England Biolabs) using 1x Quick Ligase Buffer, 18uL of the blunt end repaired DNA and 0.25uM of the Adapter Mix, and incubated for 20min at 22°C. Afterwards the DNA was purified again with the MinElute Purification Kit (Qiagen), but eluting in 22uL of the EB-/Tween buffer. Adapter fill in was performed with 0.4 U Bst 2.0 DNA Polymerase (New England Biolabs) using 1x Isothermal Buffer (New England Biolabs), 0.25nM dNTP's (ThermoScientific), and balanced with Water and 20uL of the adapter ligated DNA. Incubation was for 30min at 37°C followed by 10min for 80°C.

DNA library construction and high-throughput sequencing

Historic samples

The DNA concentration of each Radcliffe sample was determined with a Qubit fluorometer using the dsDNA high sensitivity assay, and 100 ng of DNA was used to build libraries. A library blank control was processed simultaneously with the DNA samples. Libraries were built with a protocol based on [55], and had one internal index and one external index. Blunt ends were repaired by incubating 100 ng DNA at 25_oC x 15 min followed by 12_oC for 5 min with the following master mix: 1X Tango Buffer (ThermoFisher), 100 uM dNTPs, 1 mM ATP, 0.5 U/uL T4 polynucleotide kinase, 0.1 U/uL T4 DNA polymerase, balance dH_2O. Blunt-end repaired samples were then cleaned with a QIAGEN MinElute kit as follows: samples were diluted in 350 uL buffer PB, and mixed by pipetting. Samples were spun at 6000 g for 1 min, washed with 750 uL buffer PE and spun at 10000 g for 1 min, then spun an additional 1 min to remove residual buffer. DNA was eluted by adding 21 uL buffer EB, incubating samples at room temperature for 5 minutes, then followed by centrifugation at 13000 g for 1 min. Adapters with internal indexes were ligated to the samples as follows. Adapter ligation master mix was prepared with the following concentrations: 1X T4 DNA ligase buffer, 5% PEG-4000, 2.5 uM adapter mix, 0.125 U/uL, balance water. An equal volume of adapter ligation mix was added to the eluate from blunt end repair and incubated at 22_oC x 30 min. Libraries were cleaned by adding all 40 uL to 200 uL buffer PB, mixing, and transferring the entire volume to a QiaQuick column. Columns were spun at 6000 g for 1 min and again at 10000 g for 1 min. DNA was eluted by adding 21 uL buffer EB, incubating samples at room temperature for 5 minute, followed by centrifugation at 13000 g for 1 min. Adapters were filled in by adding an equal volume of the following master mix to the adapter ligation eluate and incubating at 37_oC x 20 min: 1X ThermoPol reaction buffer, 250 uM each 4 dNTP, 0.3 U/uL Bst polymerase (large fragment), balance H_2O. The Bst enzyme was inactivated by incubation at 80_oC for 20 min, and samples were stored at -20_oC.

The single-index library samples were then double indexed using PCR amplification. A calibration RT-PCR was set up for each sample. The number of cycles used for PCR amplification of each library was chosen by selecting two cycles after the amplification plot reached plateau. A PCR for library amplification was prepared and run for each sample as above, but using the indexing primer with the appropriate external index. Each amplified library was then checked with an Agilent Tape Station to determine the insert size range and concentration. The AccuPrime PFX-generated libraries were pooled at equimolar concentrations into 4 separate groups, while the KAPA HiFi Uracil+-generated libraries were pooled into a single group at equimolar concentrations. Pooled libraries were cleaned with a QIAGEN QiaQuick kit and then size-selected using a PippinPrep, keeping only libraries in the size range of 150-400bp. The size-selected libraries were run on the Tape Station to check fragment length distribution and concentration and then sent for sequencing. All samples were sequenced on an Illumina HiSeq2500 using 150bp paired-end RapidRun chemistry at the University of Copenhagen National High-Throughput DNA Sequencing Centre.

Modern calculus

For the modern calculus samples, a 1:50 dilution of the libraries were produced to quantify with the IS7 and IS8 Primers in a quantitative Real-time PCR(qPCR; DyNamo SYBR Green qPCR Kit; ThermoScientific) on a LightCycler 48 (Roche). The libraries were split in 1*10^10 Copies of DNA for the following indexing step. With the PfuTurbo DNA Polymerase (Agilent), the libraries were double indexed (Meyer and Kircher 2010 Cold spring Harbour Protocols, Kircher et al. 2012 Nucleic Acid Research). Libraries were amplified in a 100uL reaction using 1x Pfu Turbo Buffer (Agilent), 0.3mg/ml BSA (New England Biolabs), 0.38mM dNTP's (Thermo Scientific), 0.025 U of the Pfu Turbo Polymerase (Agilent) and 0.2 uM (each) of the specific P5 and P7 Primer, again balancing with Water. Incubation was for 5min at 95°C, followed by 10 cycles of 1min at 95°C, 30 sec at 58°C, 1min at 72°C, ended with an elongation for 10min at 72°C.The reactions were cleaned up with the MinElute Purification Kit as above, eluting in 50uL of EB Buffer (Qiagen) containing 0.05% Tween 20 (Sigma-Aldrich).With a 1:1000 dilution, the libraries were quantified with the IS5 and IS6 Primers with the qPCR method (see above).

Afterwards the nM was checked by TapeStation with the D1000- Kit (Agilent). An equimolar Pool of the libraries (blanks separately) of 10nM each library were prepared. Shotgun sequencing was performed on a NextSeq500 platform from Illumina with 40 Million reads per sample and 2 Million reads per blank.

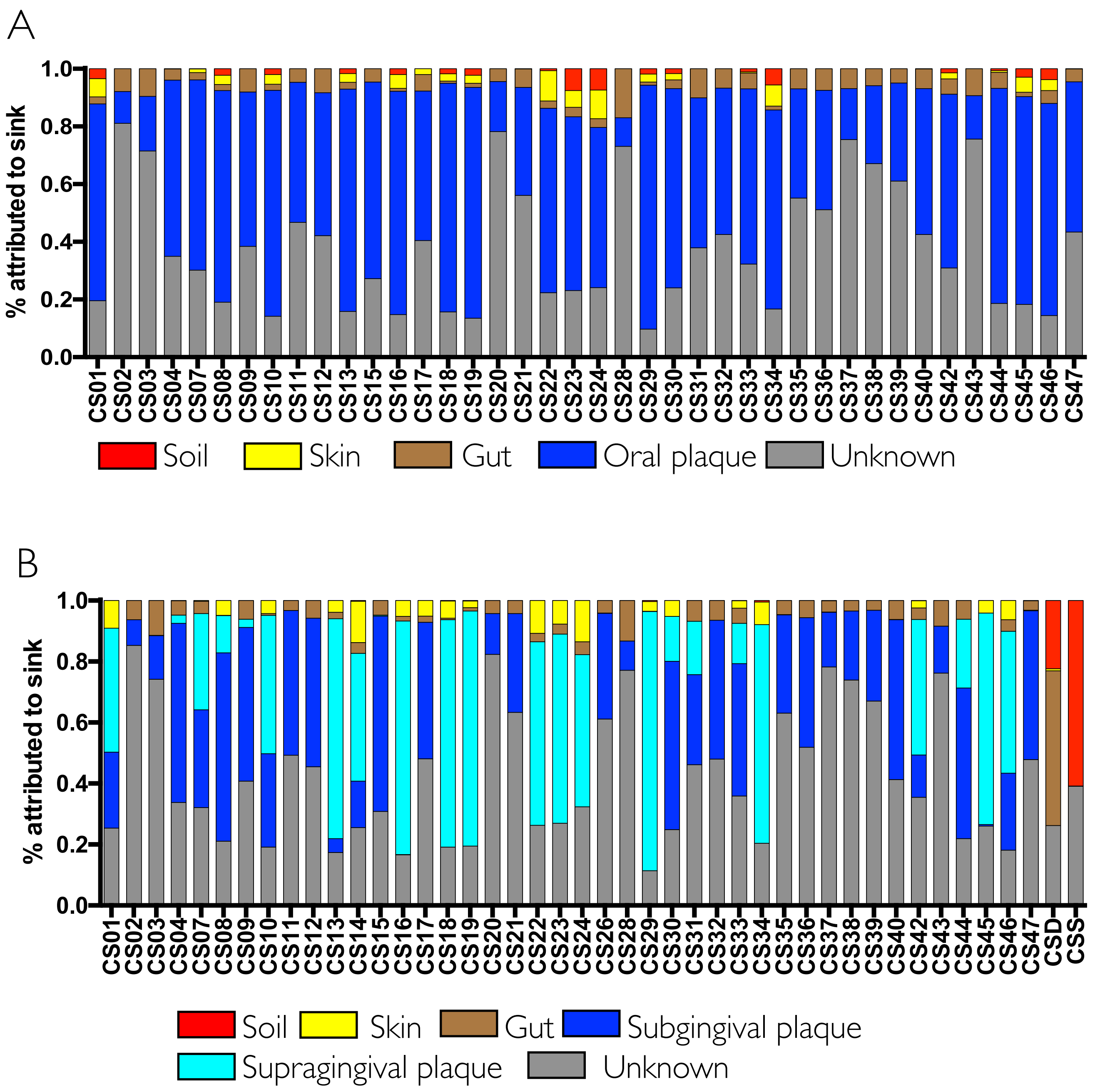

Figure S1. SourceTracker analysis of Radcliffe dental calculus samples. A. SourceTracker source proportion estimates of all calculus samples included in the analysis rarefied to 5500 reads, with supra- and subgingival plaque sources combined as "oral plaque". B. SourceTracker source proportion estimates of all calculus samples plus the dentin and soil sample rarefied to 758 reads to enable inclusion of the soil sample. The calculus samples show a predominantly oral plaque signature, while the dentin and soil samples show predominantly gut and soil signatures, respectively.

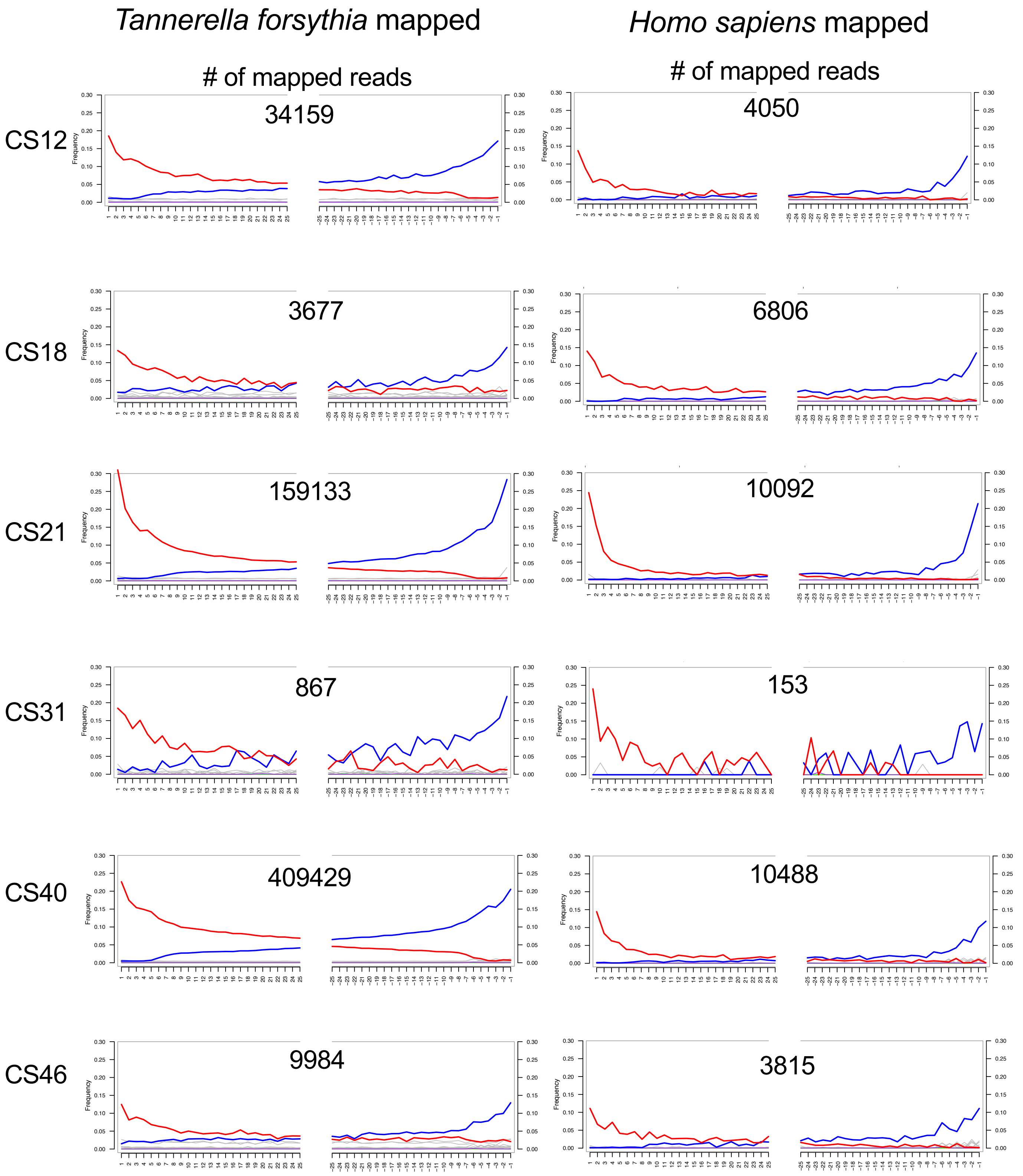

Figure S2. DNA damage patterns authenticate historic calculus samples. MapDamage plots reveal cytosine to thymine transitions at the ends of molecules and breakpoints coinciding with depurination. The 6 samples shown are those for which we have DNA, protein, and metabolite data. Smoothness of curves is related to the number of reads that mapped. Left-panel mapDamage plots of reads mapped to the *Tannerella forsythia* genome. Right-panel mapDamage plots of reads mapped to the *Homo sapiens* genome.

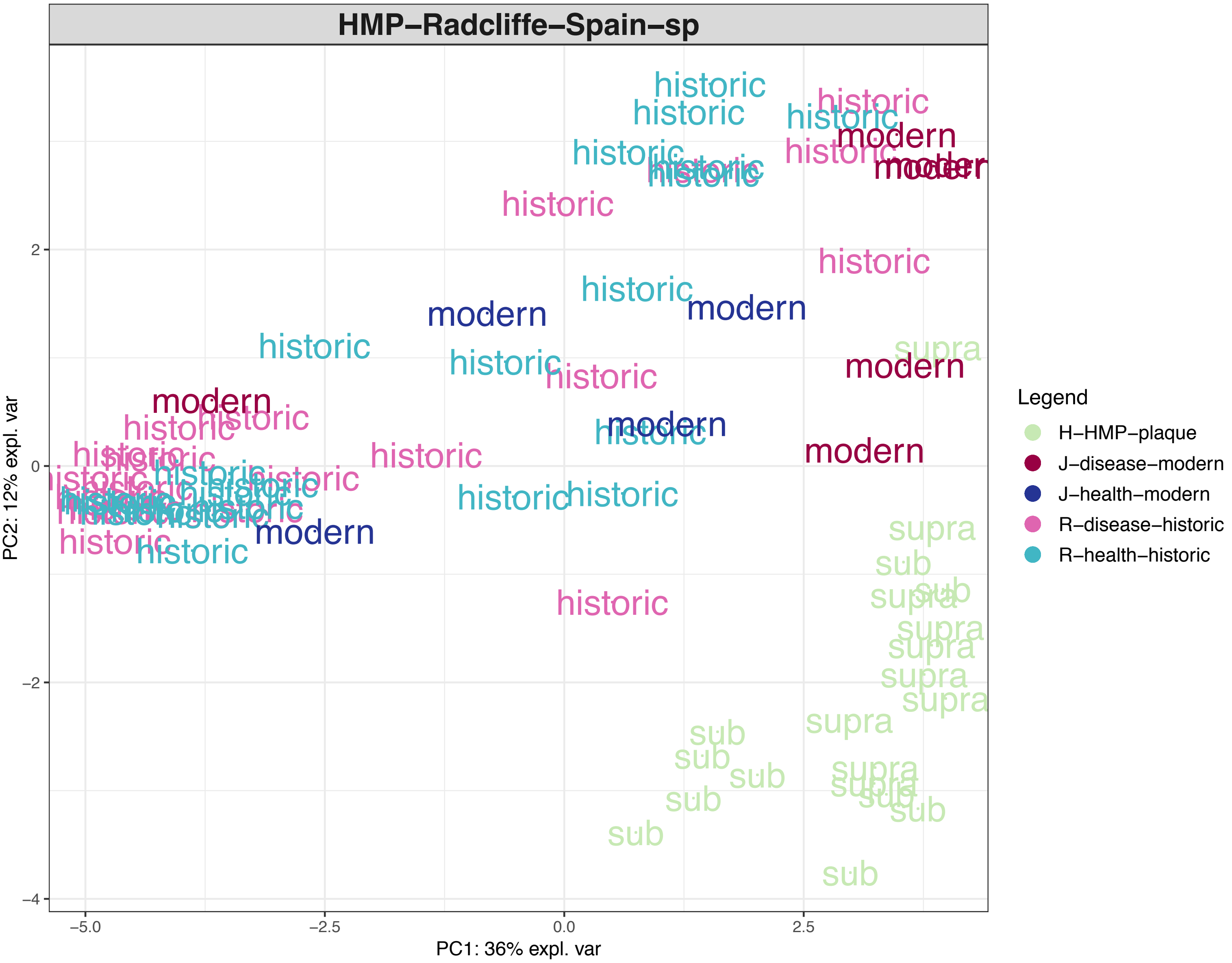

Figure S3. Dental plaque and dental calculus contain distinct microbial communities. Same as Figure 1B but with the calculus samples named by time period and the plaque samples named by source (supra-gingival or sub-gingival)

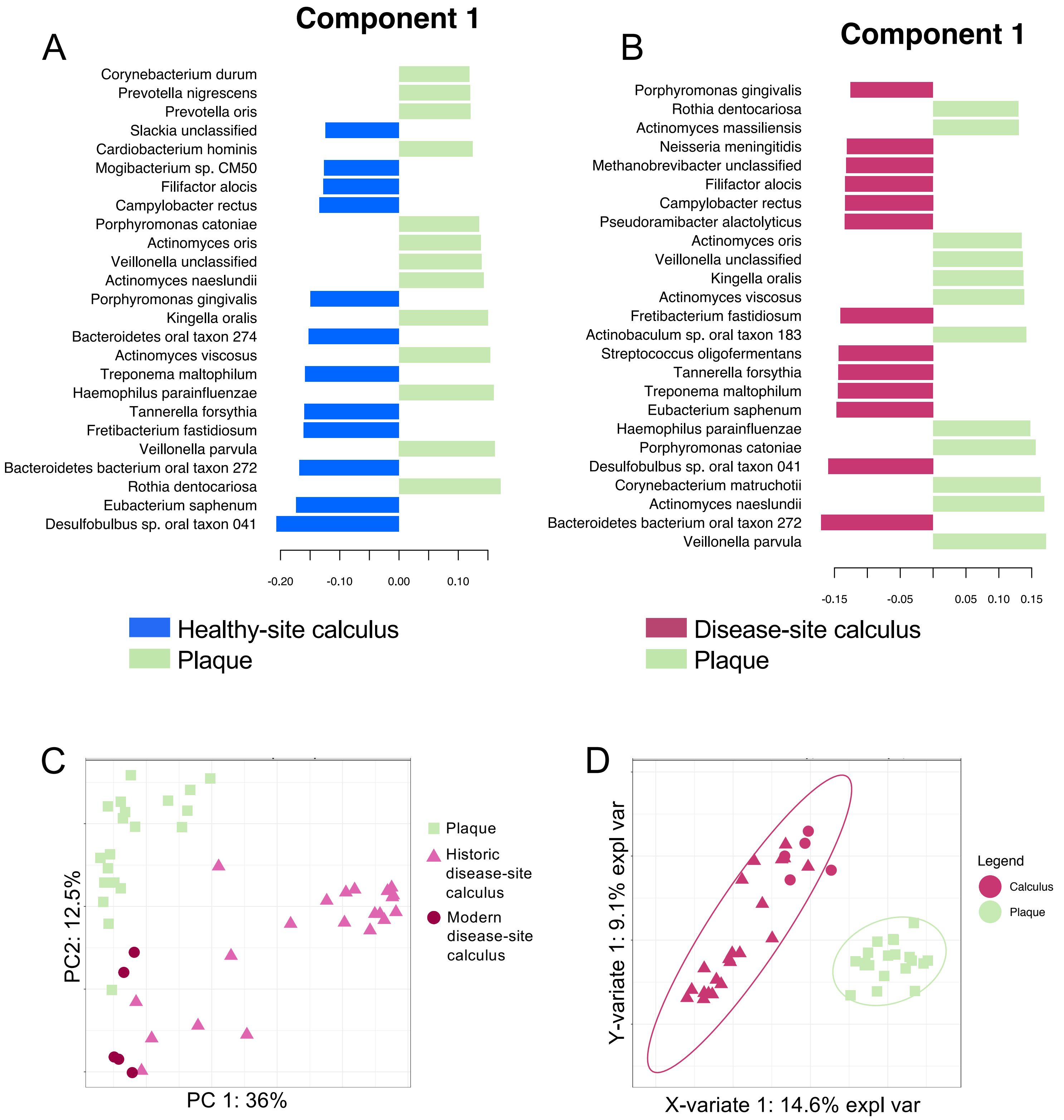

Figure S4. A. Taxa that contribute to discriminating plaque and all healthy-site calculus samples along component 1 of the sPLS-DA. B. Taxa that contribute to discriminating plaque and all disease-site samples along component 1 of the sPLS-DA. C. PCA as in Figure 2D but without the outlier modern disease-site sample. D. sPLS-DA as in Figure 2F but without the outlier modern disease-site sample. Both C and D are visually highly similar to 2D and 2F, and the variance explained by the 1st 2 components are highly similar.

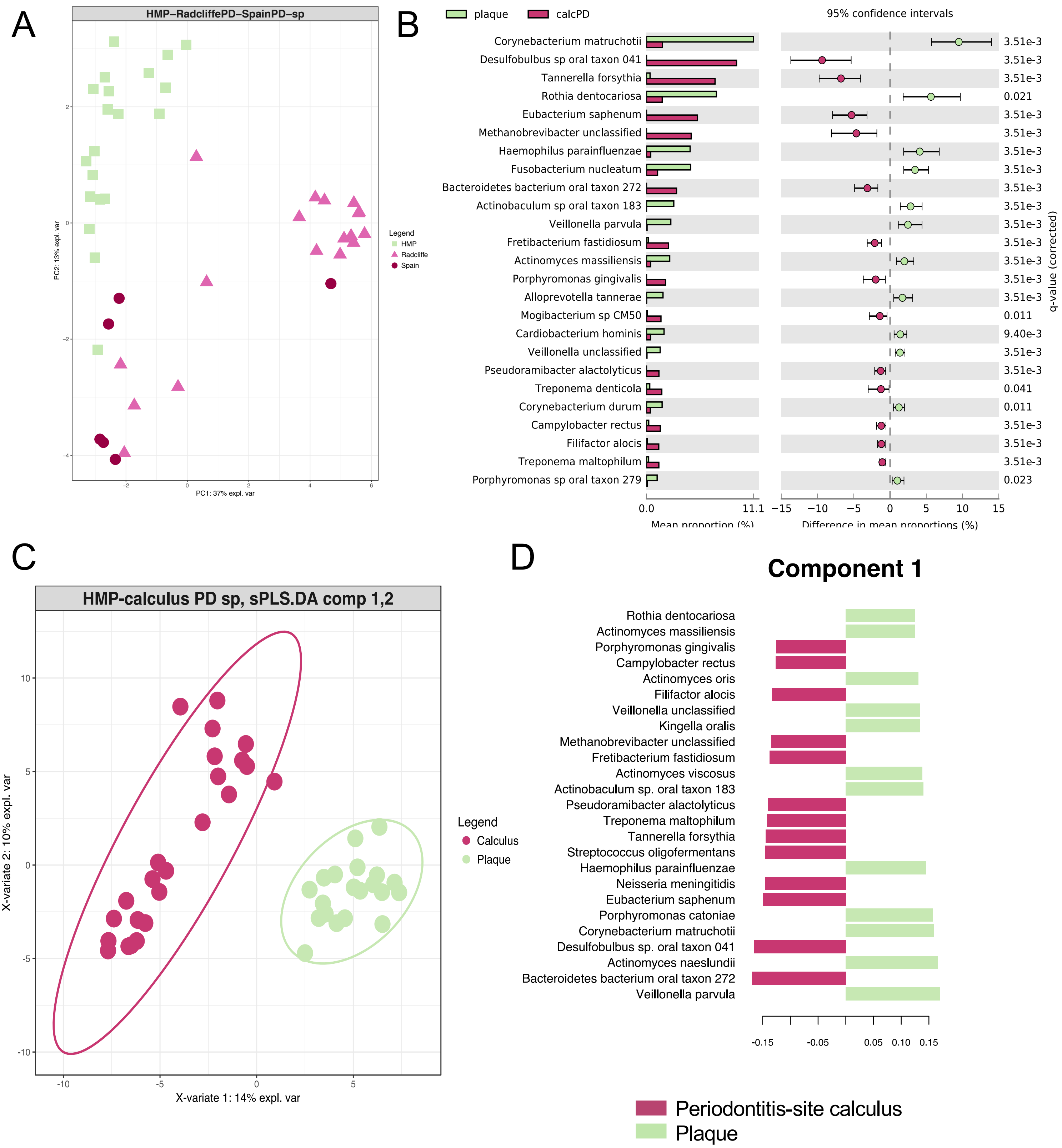

Figure S5. Periodontal disease-site calculus and healthy-site plaque contain distinct microbial profiles. A. Principal components analysis (PCA) of plaque and historic periodontal disease-site calculus cluster samples by type. B. Distinct species are significantly more abundant in plaque and historic periodontal disease-site calculus. C. Microbial profile differences between plaque and historic periodontal disease-site calculus are sufficient for discrimination of sample types by sparse partial least squares discriminant analysis (sPLS-DA). D. Taxa that contribute to discriminating plaque and periodontal diseaes-site calculus samples along component 1 of the sPLS-DA.

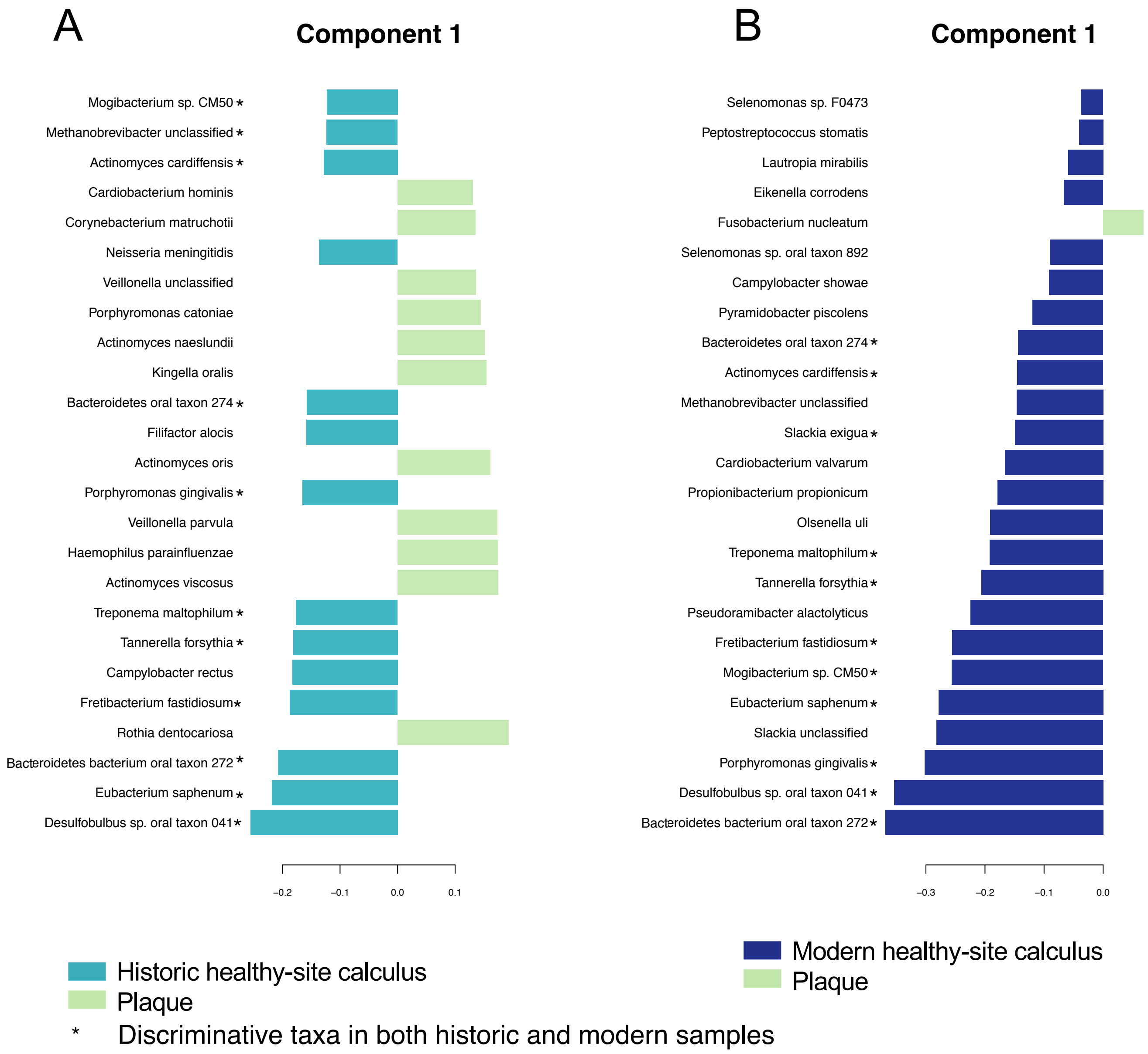

Figure S6. Taxa that contribute to discriminating sample types in from sPLS-DA. A. Taxa with significant differential abundance between plaque and healthy-site historic calculus. B. Taxa that contribute to discriminating plaque and modern healthy-site calculus samples along component 1 of the sPLS-DA.

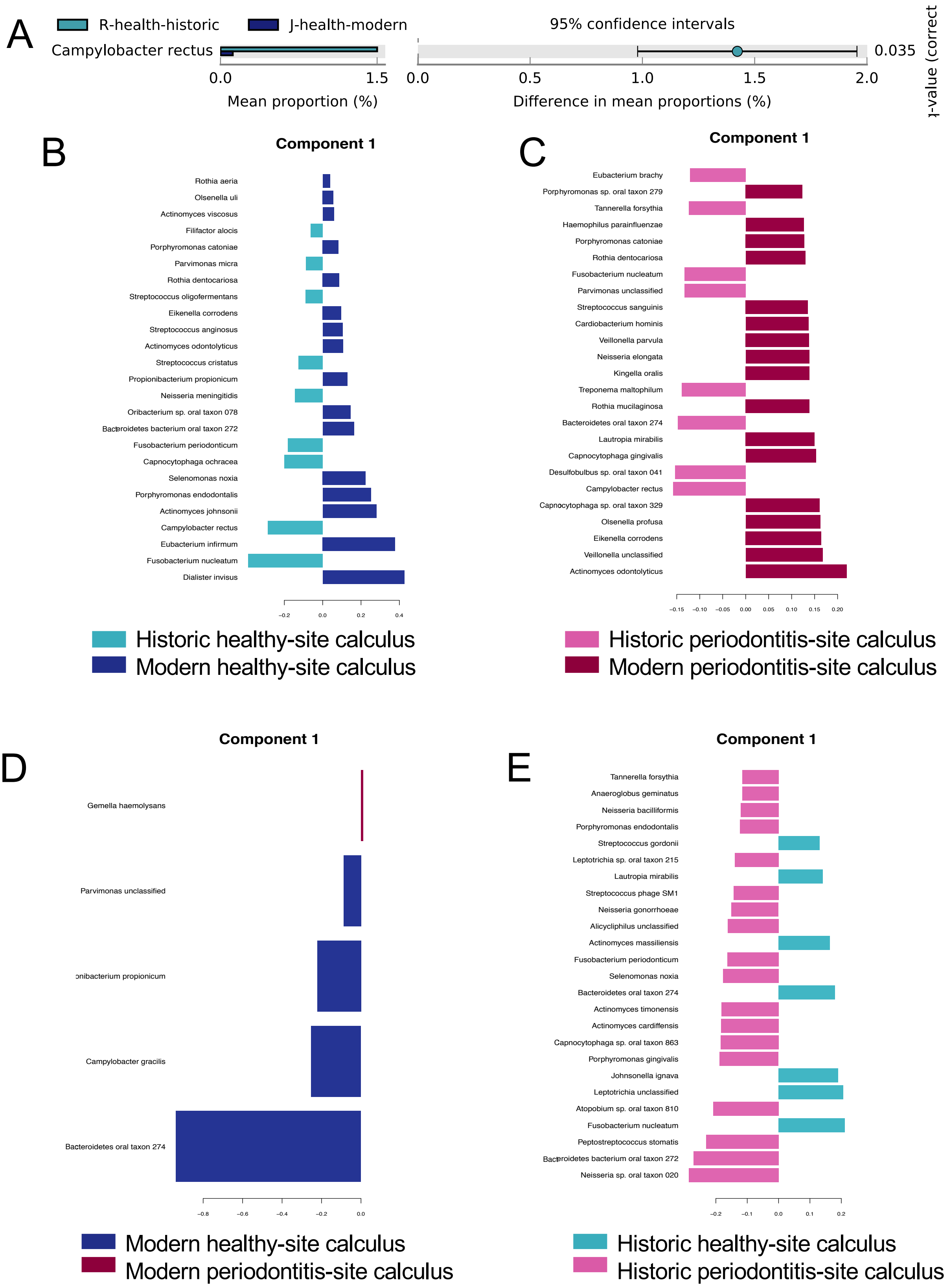

Figure S7. Taxa that contribute to discriminating sample types from sPLS-DA. A. Taxa with significant differential abundance between healthy-site modern and historic calculus. B. Taxa that contribute to discriminating modern and historic healthy-site samples along component 1 of the sPLS-DA. C. Taxa that contribute to discriminating modern and historic disease-site samples along component 1 of the sPLS-DA. D. Taxa that contribute to discriminating modern healthy- and disease-site samples along component 1 of the sPLS-DA. E. Taxa that contribute to discriminating historic healthy- and disease-site samples along component 1 of the sPLS-DA.

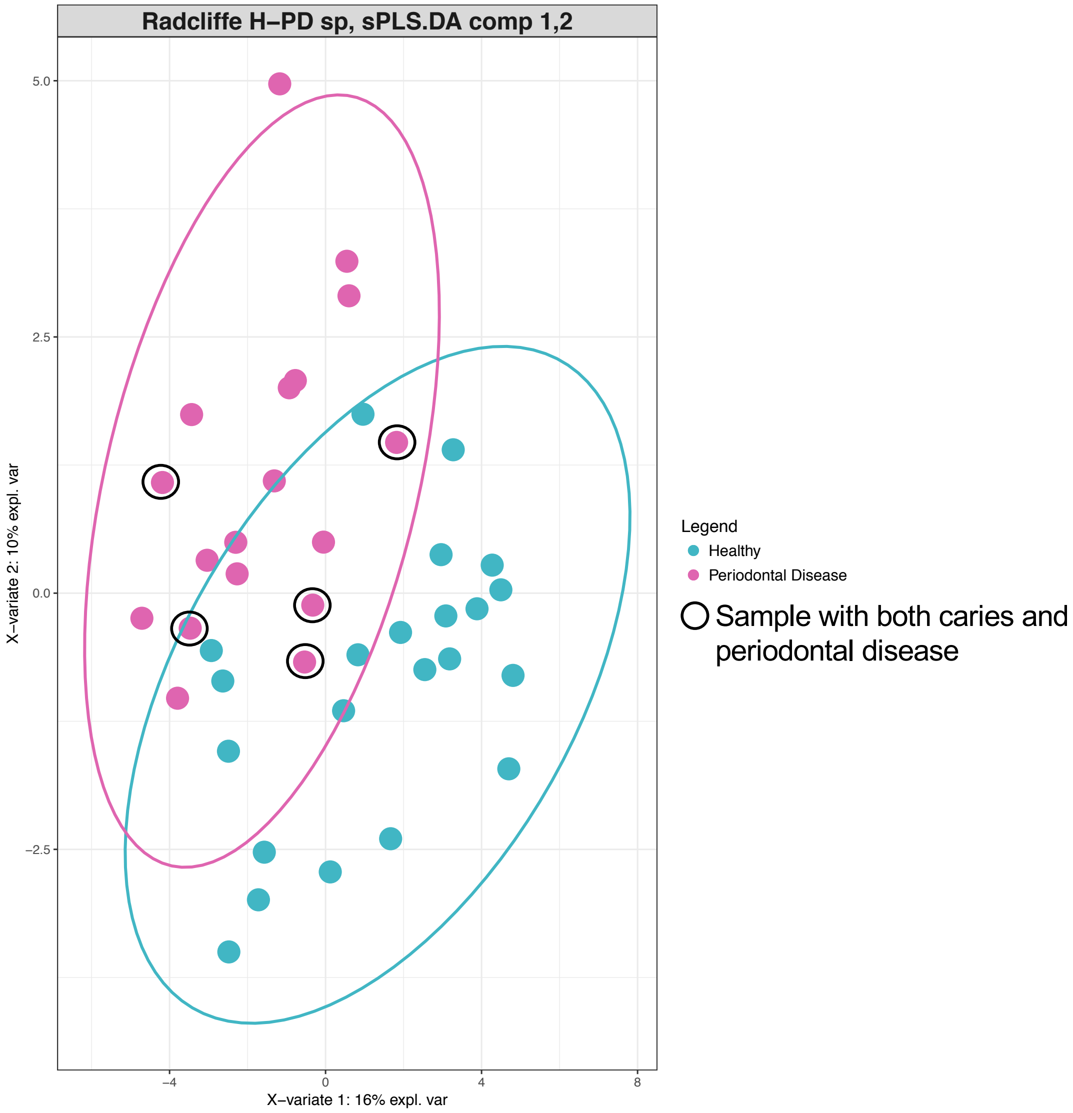

Figure S8. sPLS-DA of the Radcliffe healthy site vs. periodontal disease-affected site samples. Same as Figure 4H but with the samples from sites affected by both periodontal disease and caries circled.

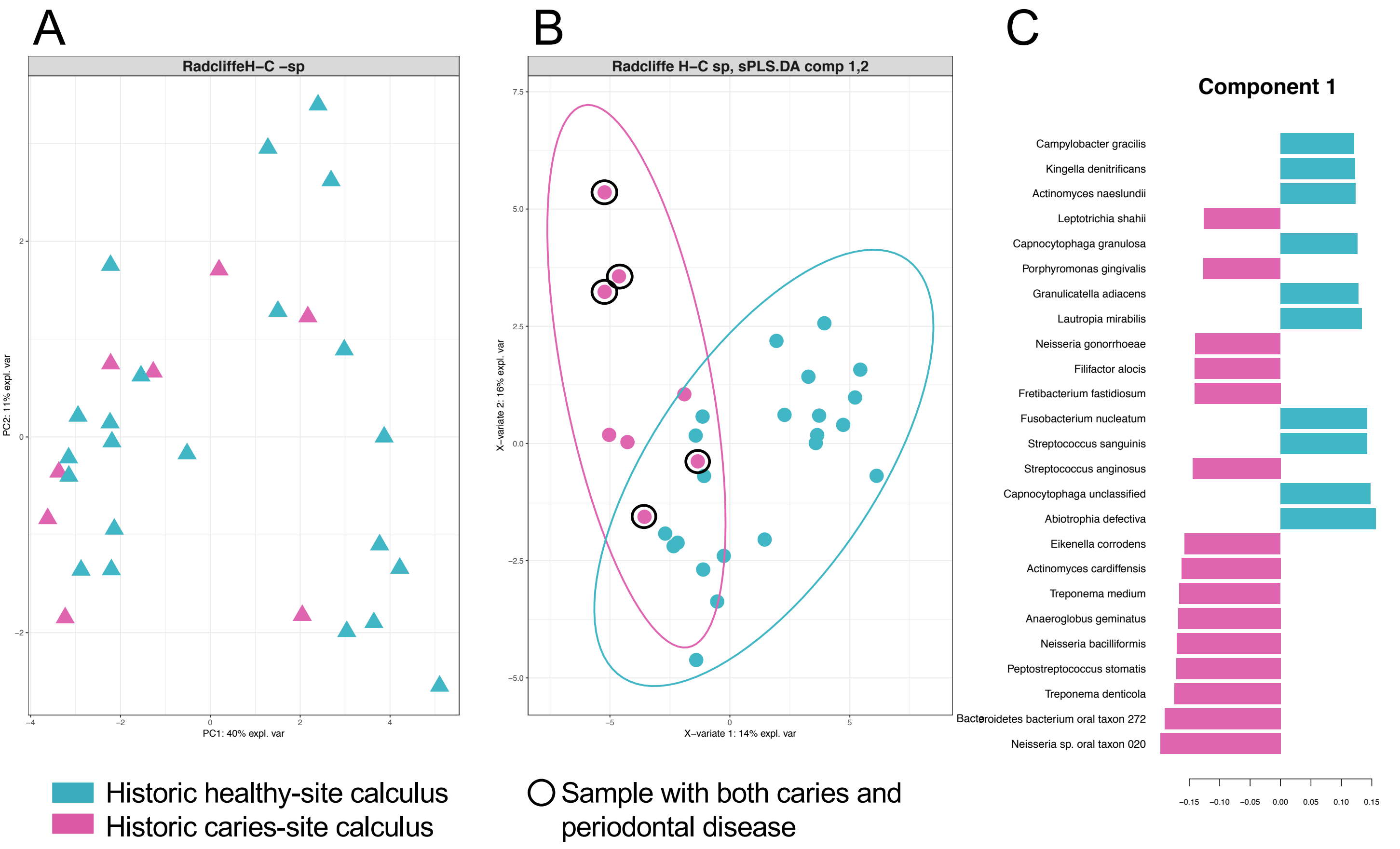

Figure S9. Historic healthy- and caries-site calculus microbial community profiles overlap. A. Principal components analysis (PCA) does not cluster historic healthy- and caries-site calculus distinctly. B. Historic healthy- and caries-site calculus do not contain sufficient discriminatory taxa for distinct clustering by sparse partial least squares-discriminant analysis (sPLS-DA). Samples with a black ring are from teeth with both caries and periodontal disease. C. Taxa that contribute to discriminating healthy- and caries-site samples along component 1 of the sPLS-DA.

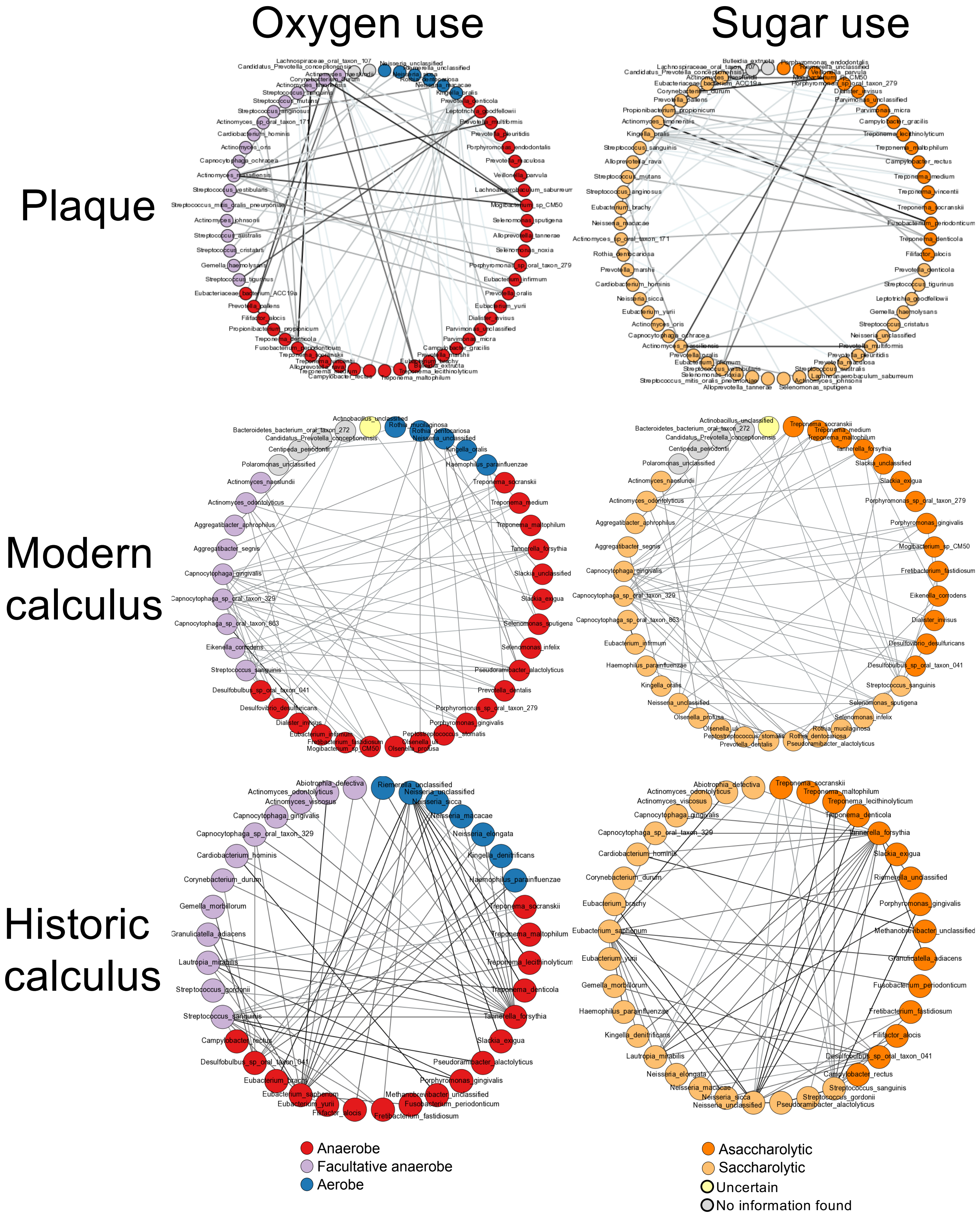

Figure S10a. Same as Figure 5, but with species names.

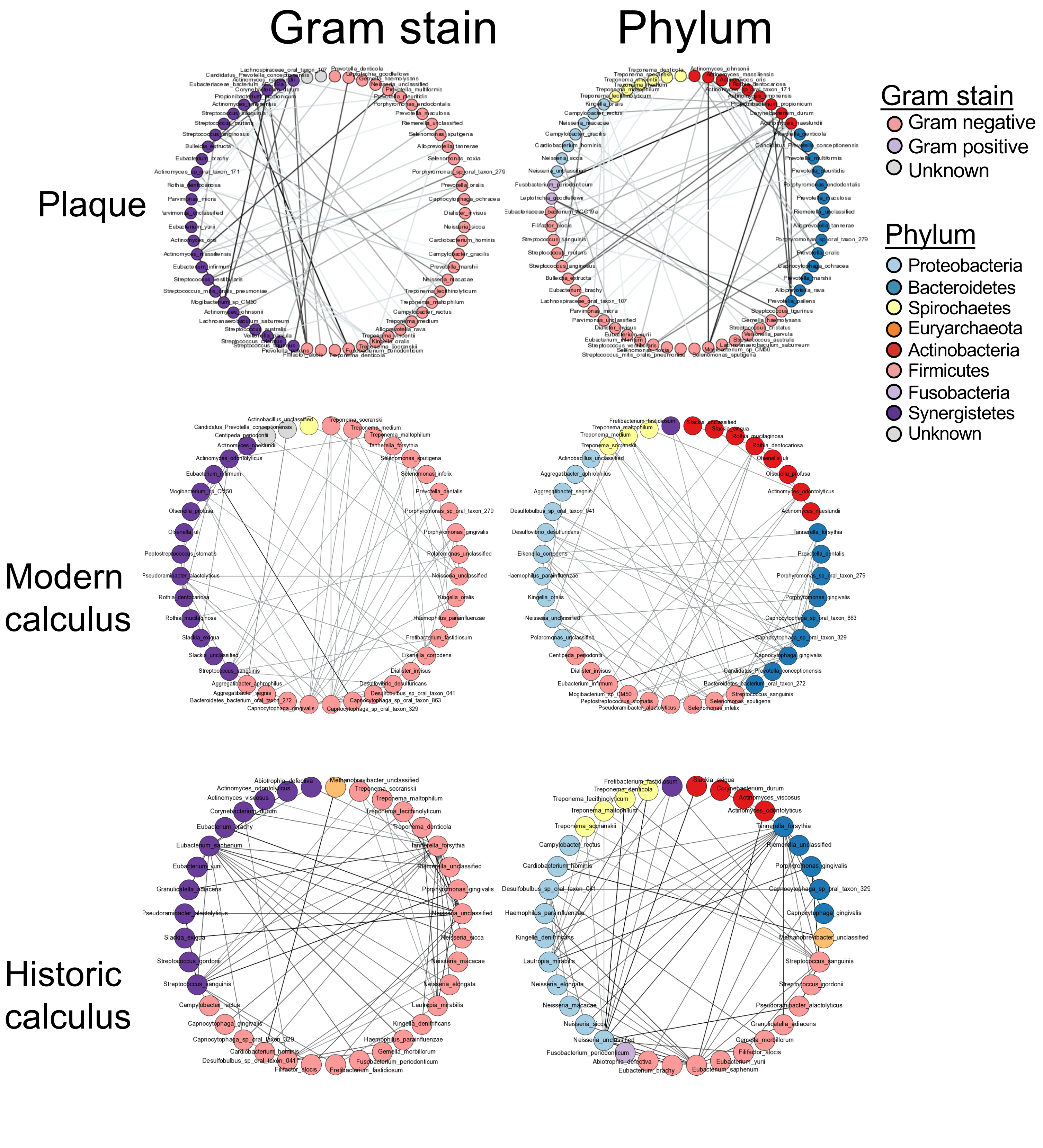

Figure S10b. Microbial co-exclusion patterns do not reflect cell wall structure or phylum-level taxonomy. Network graphs presenting species as nodes and co-exclusion between species as edges, where darker lines indicate stronger co-exclusions. Nodes are colored based on cell wall structure (Gram stain) (left) and phylum (right). Patterns of co-exclusion based on cell wall structure or phylum are not evident.

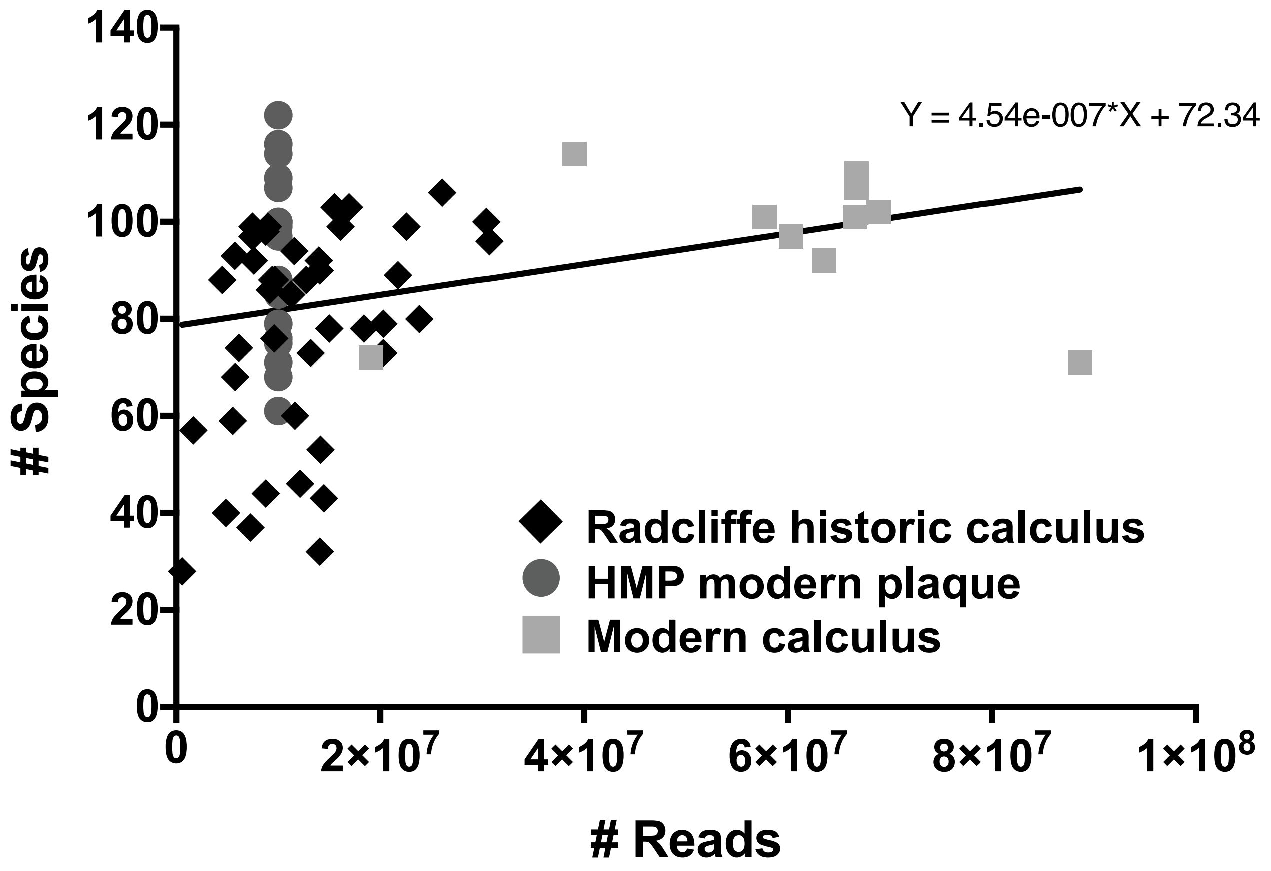

Figure S11. The number of sequencing reads in each sample vs. the number of species detected has a slight positive correlation.

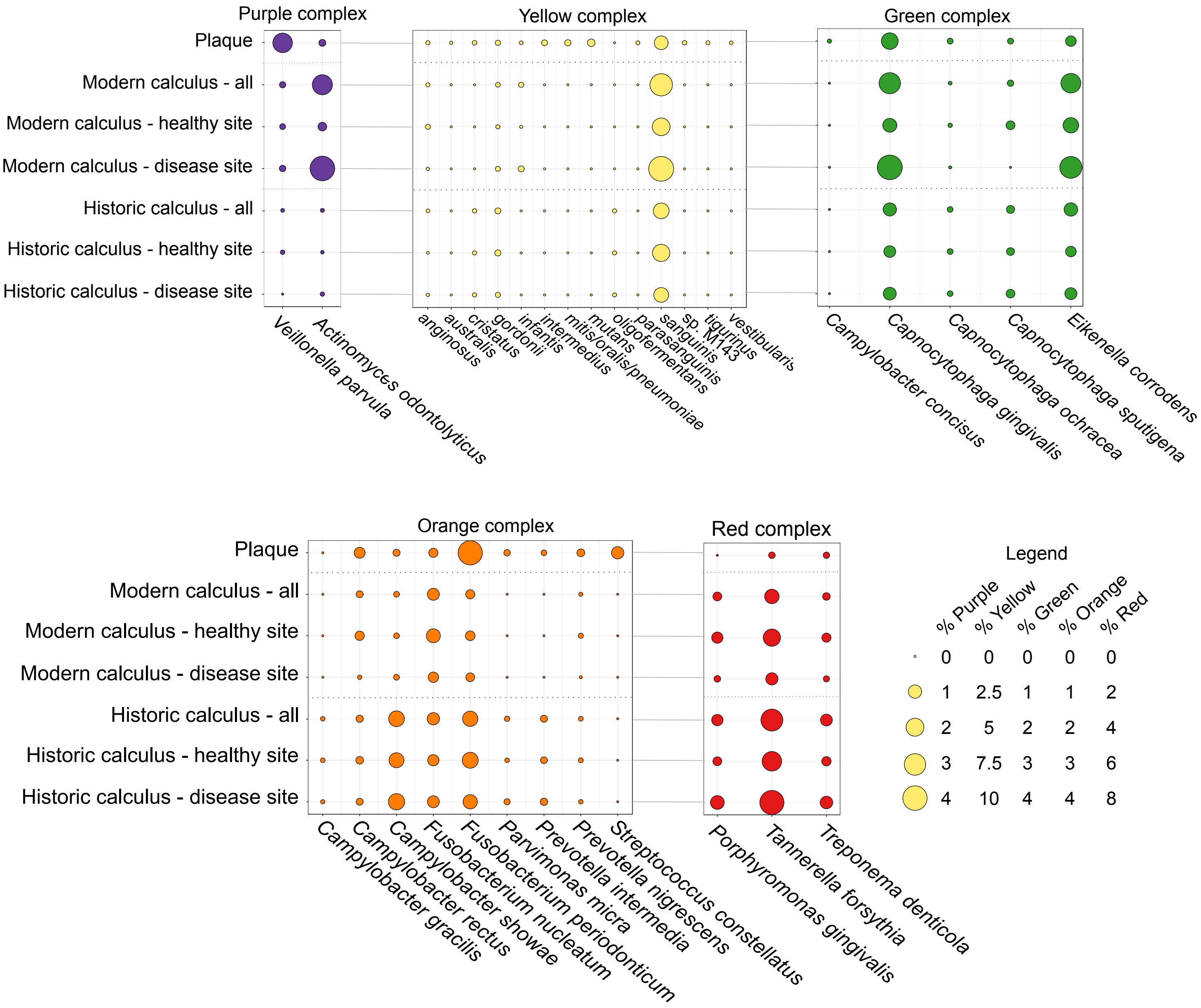

Figure S12. Bubble charts presenting the relative abundance of species comprising Socransky's complexes in plaque and calculus, separated by health status. All species in the Yellow complex are in the genus *Streptococcus*. The relative abundance of species are summed for each sample category in Figure 6.

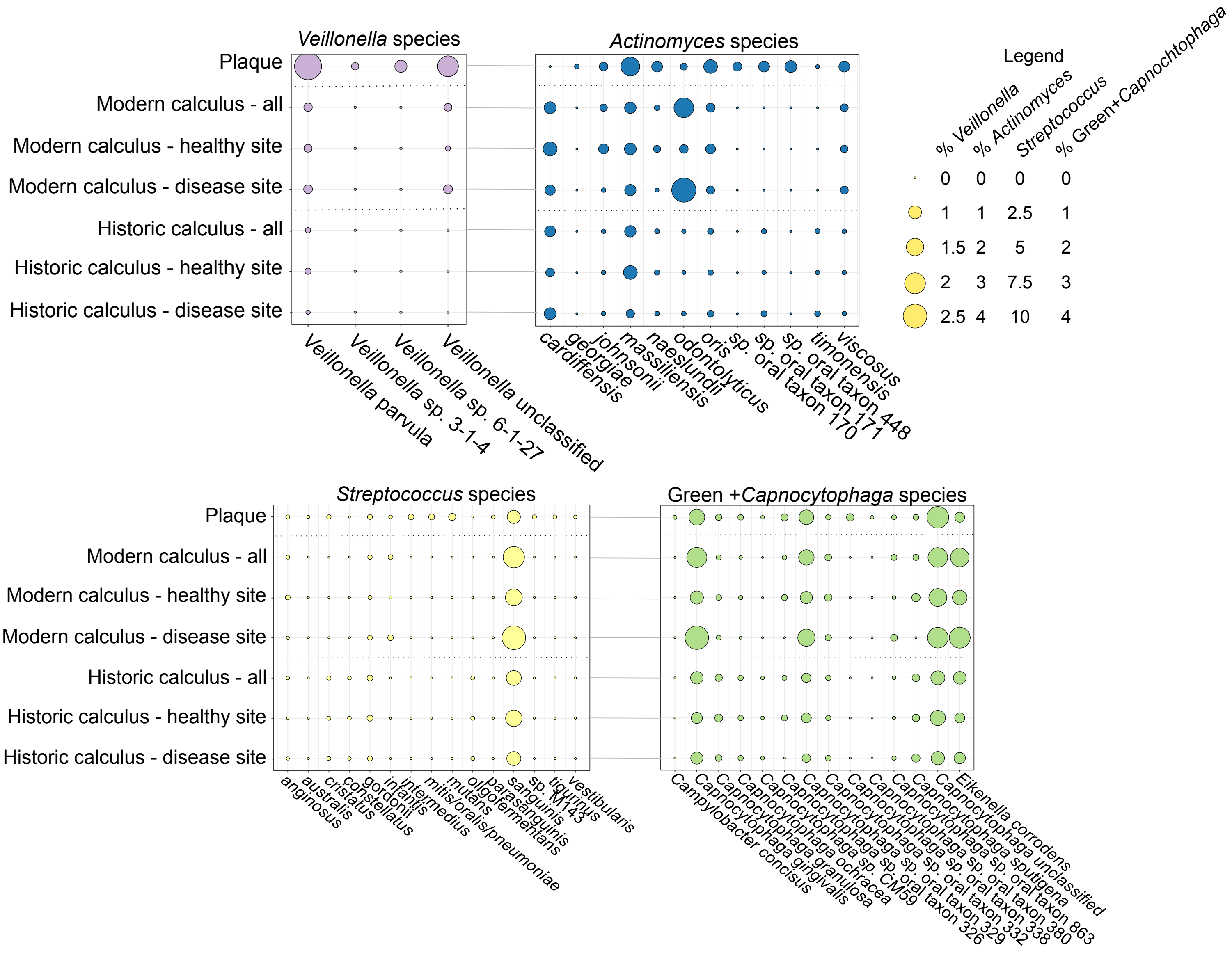

Figure S13. Bubble charts presenting the relative abundance of select species in plaque and calculus, separated by health status. The relative abundance of species are summed for each sample category in Figure 6.

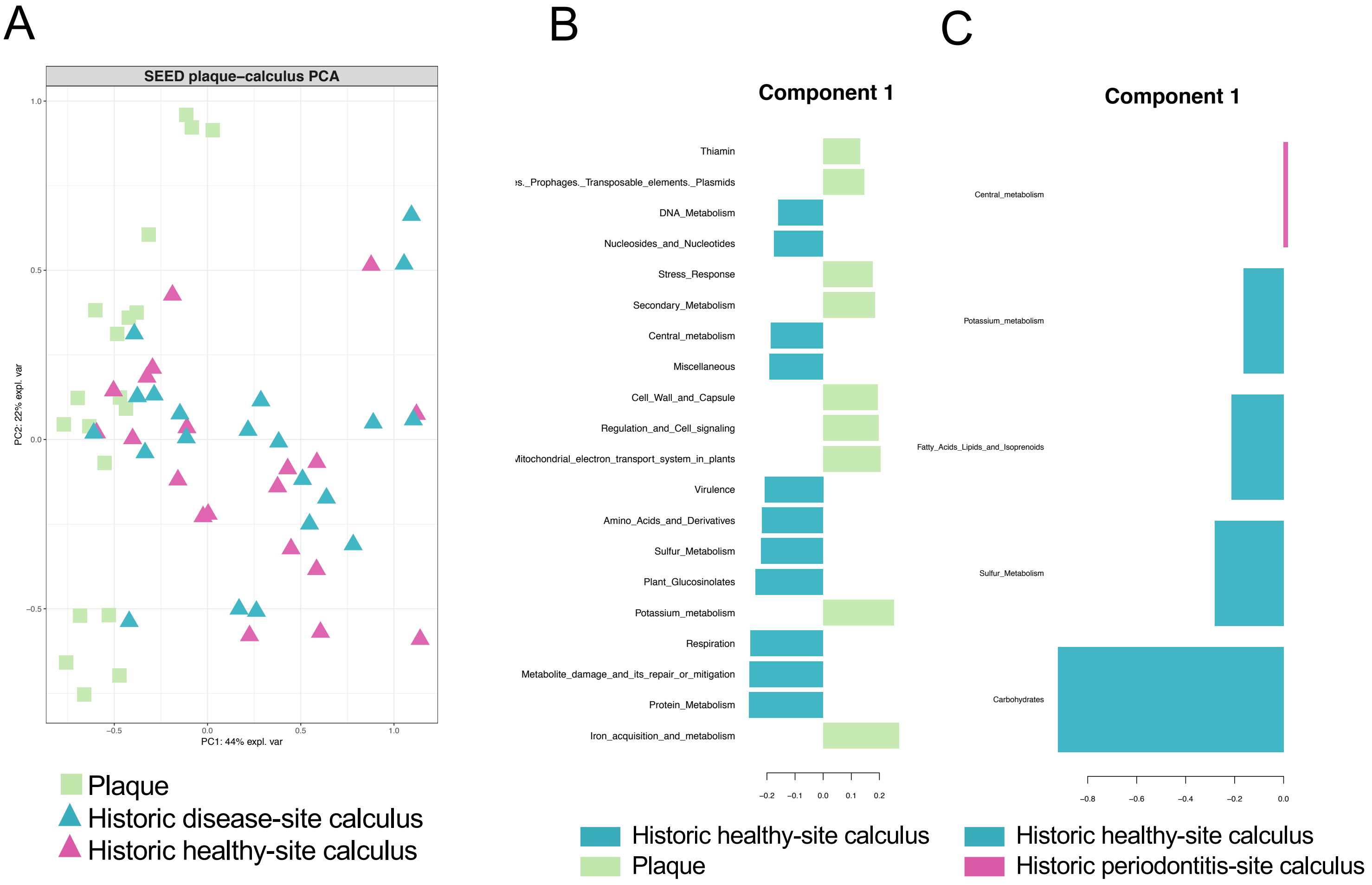

Figure S14. Functional metabolic profiles of plaque and calculus. A. Principal components analysis of SEED categorization of plaque and historic calculus with historic calculus samples colored by health status. No clustering based on health status is seen. B. SEED categories contributing to classification of sample type (plaque vs. historic healthy-site calculus) along component 1 from sPLS-DA. C. SEED categories contributing to classification of sample type (Historic healthy-site vs. periodontitis-site calculus) along component 1 from sPLS-DA.

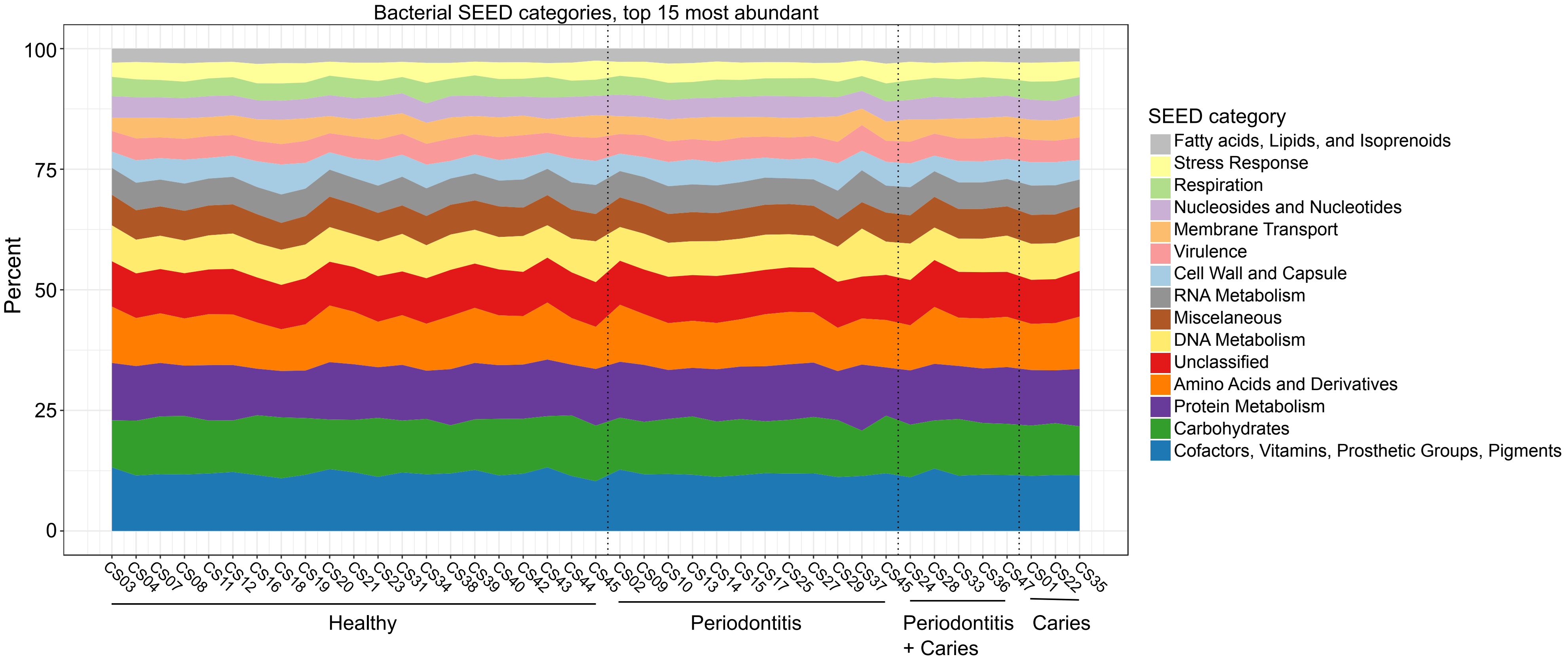

Figure S15. Relative proportion of the top 15 most abundant SEED categories in all calculus samples. Functional categorization is fairly uniform across the samples despite differences in health categories.

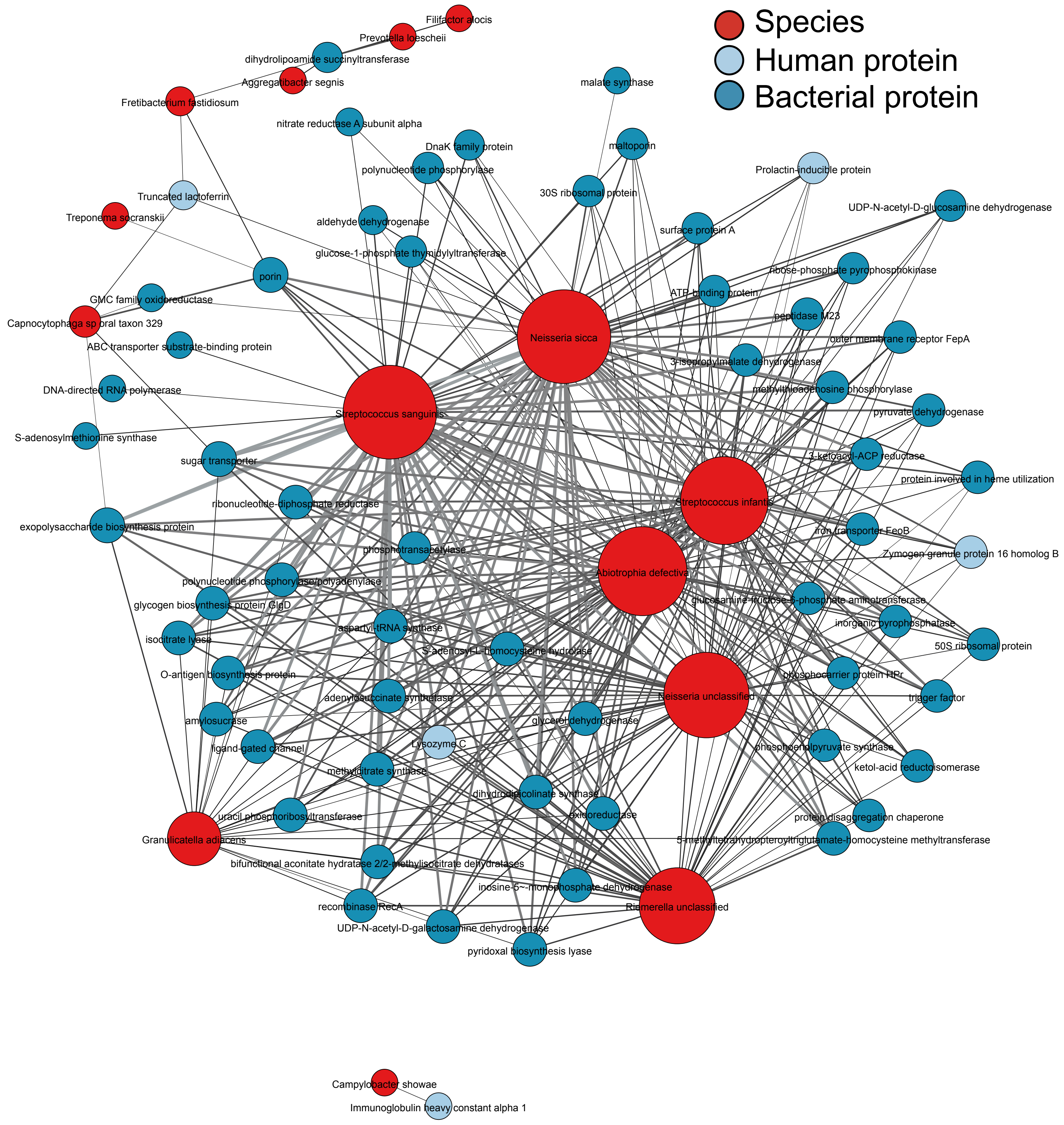

Figure S16. Network graph presenting the strongest canonical correlations (≥0.82) between species and proteins in historic dental calculus.

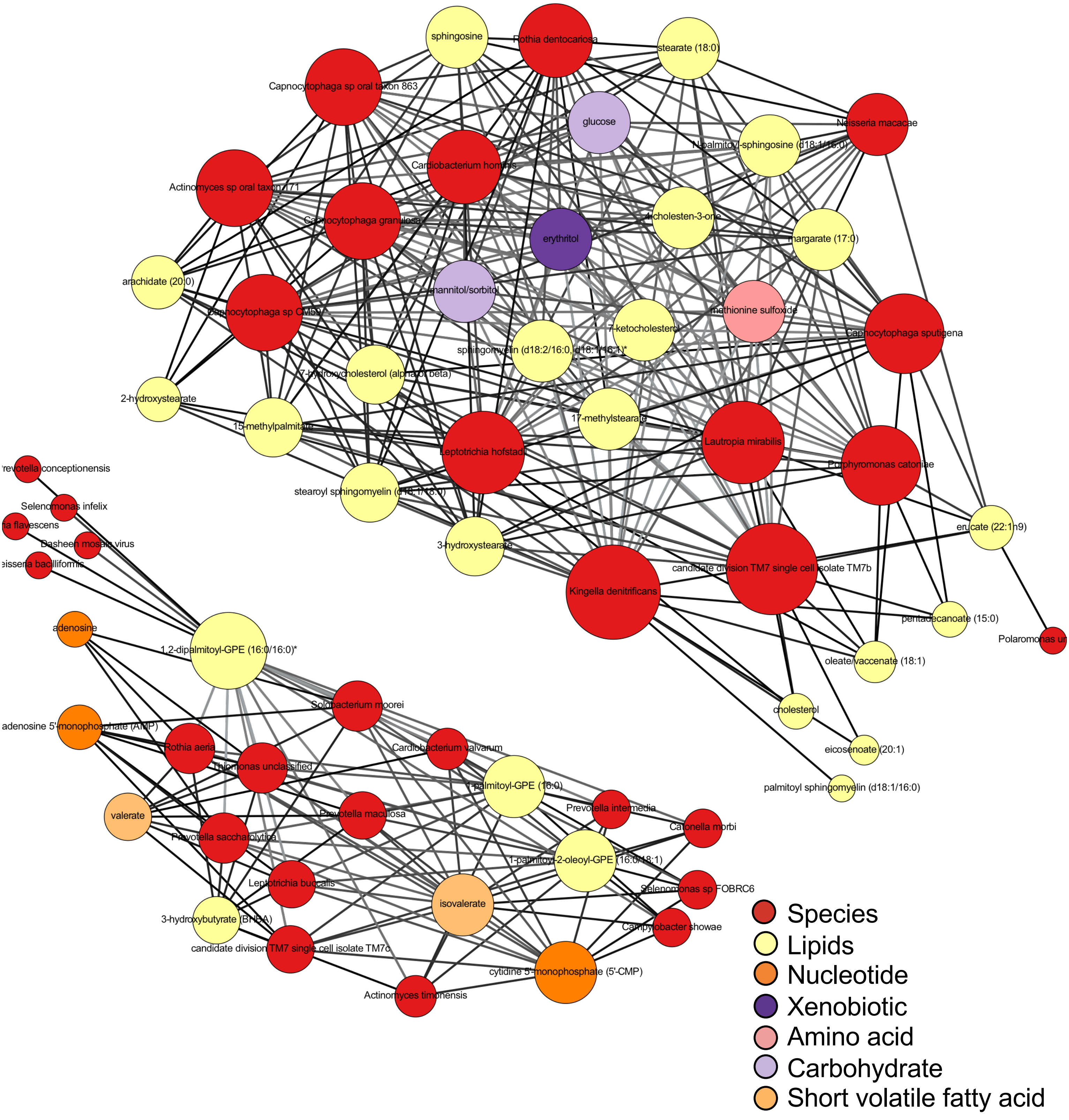

Figure S17. Network graph presenting the strongest canonical correlations (≥0.75) between species and metabolites in historic dental calculus.

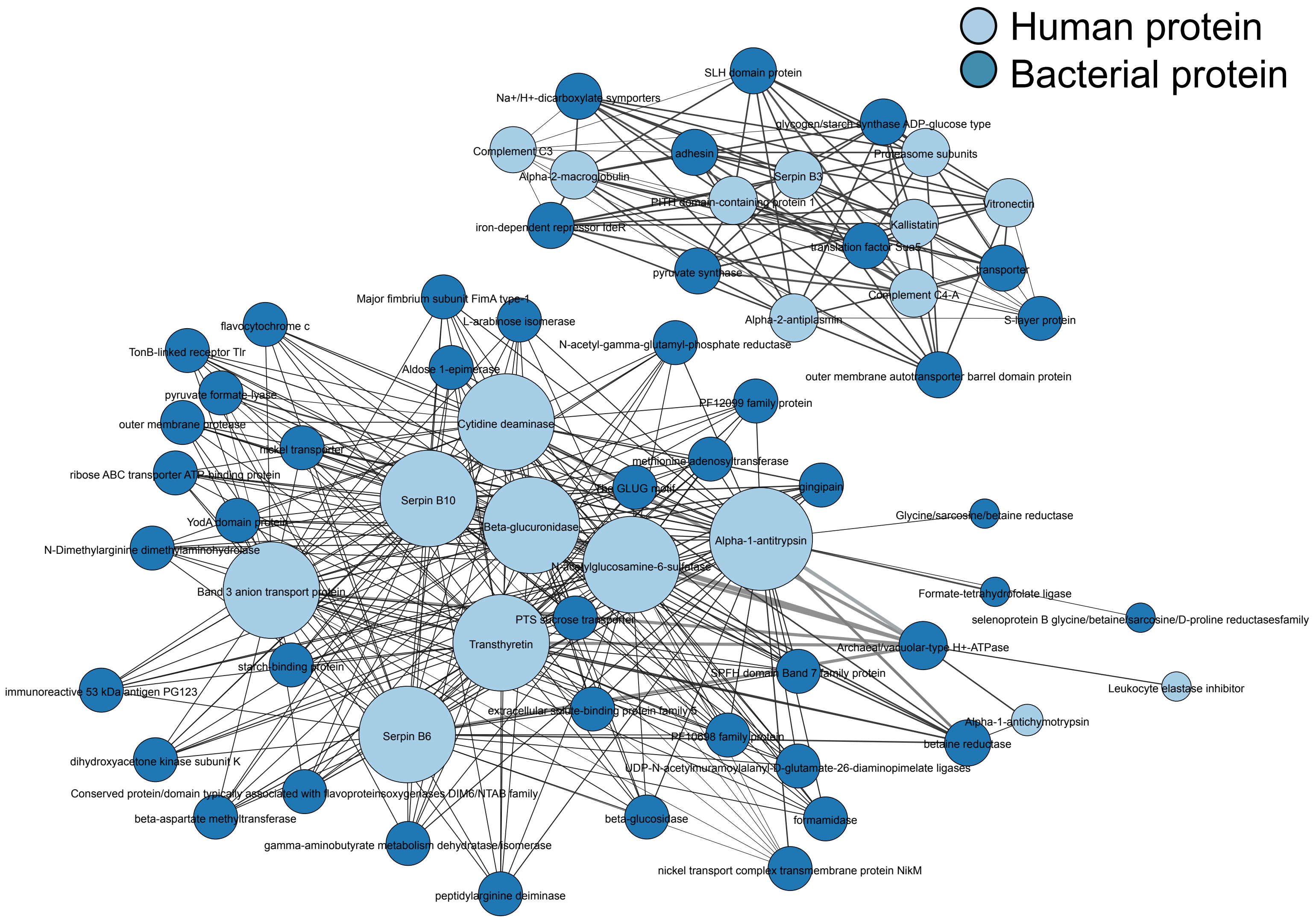

Figure S18. Network graph presenting the strongest canonical correlations (≥0.9) between human and bacterial proteins in historic dental calculus. Same as Figure 9A, but with node labels.

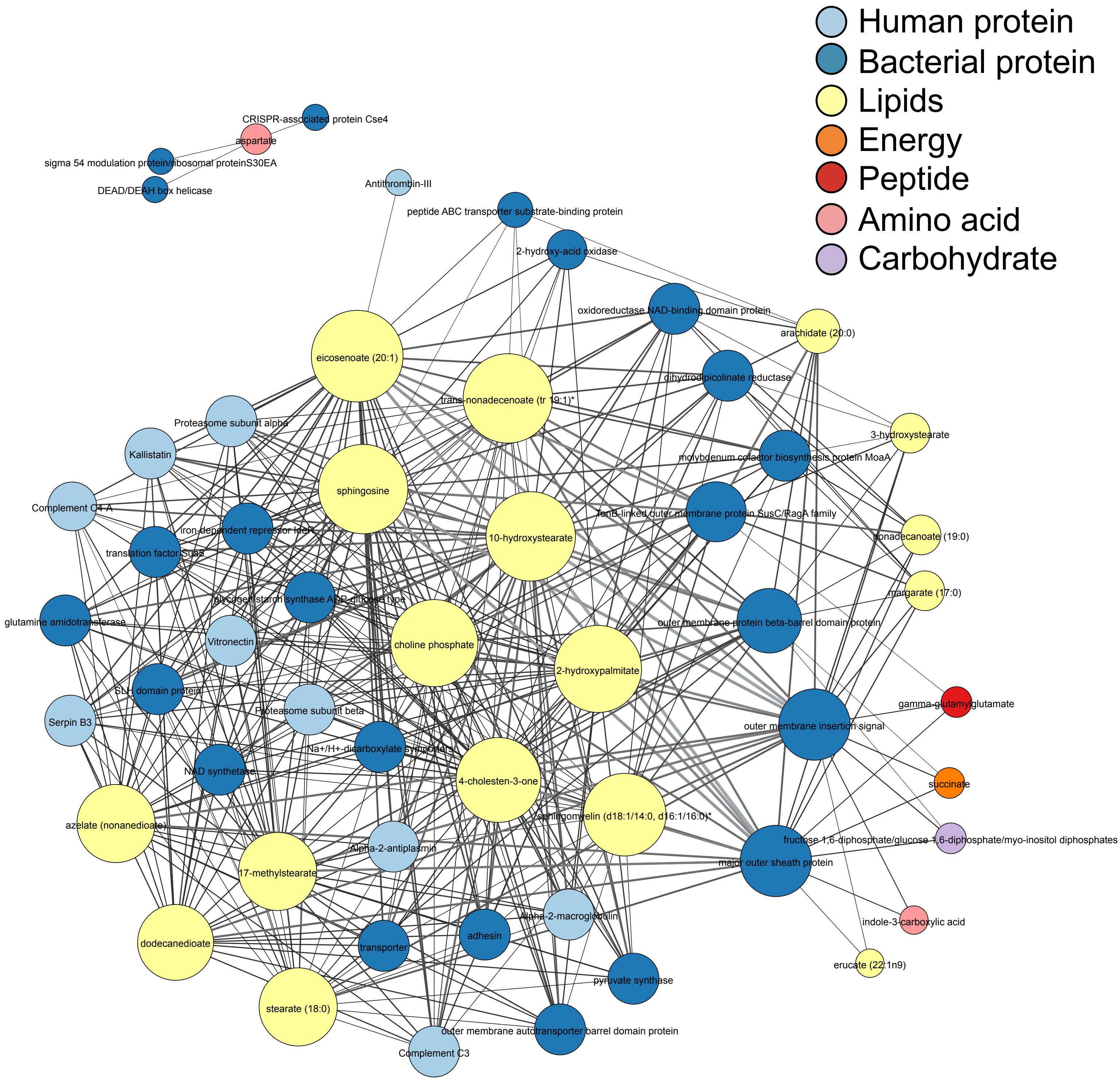

Figure S19. Network graph presenting the strongest canonical correlations (≥0.9) between proteins and metabolites in historic dental calculus. Same as Figure 9B, but with node labels.
